# Characterizing and Engineering Biomimetic Materials for Viscoelastic Mechanotransduction Studies

**DOI:** 10.1089/ten.teb.2021.0151

**Published:** 2022-08-08

**Authors:** Ludovica Cacopardo, Nicole Guazzelli, Arti Ahluwalia

**Affiliations:** ^1^ Research Center “Enrico Piaggio,” University of Pisa, Pisa, Italy.; ^2^ Department of Information Engineering, University of Pisa, Pisa, Italy.; ^3^ Centro 3R (Inter-University Center for the Promotion of the 3Rs Principles in Teaching and Research), Pisa, Italy.

**Keywords:** viscoelasticity, hydrogels, viscoelastic mechanotransduction, mechanical testing, cell mechanical memory, time scaling

## Abstract

The mechanical behavior of soft tissue extracellular matrix is time dependent. Moreover, it evolves over time due to physiological processes as well as aging and disease. Measuring and quantifying the time-dependent mechanical behavior of soft tissues and materials pose a challenge, not only because of their labile and hydrated nature but also because of the lack of a common definition of terms and understanding of models for characterizing viscoelasticity. Here, we review the most important measurement techniques and models used to determine the viscoelastic properties of soft hydrated materials—or hydrogels—underlining the difference between viscoelastic behavior and the properties and descriptors used to quantify viscoelasticity. We then discuss the principal factors, which determine tissue viscoelasticity *in vivo* and summarize what we currently know about cell response to time-dependent materials, outlining fundamental factors that have to be considered when interpreting results. Particular attention is given to the relationship between the different time scales involved (mechanical, cellular and observation time scales), as well as scaling principles, all of which must be considered when designing viscoelastic materials and performing experiments for biomechanics or mechanobiology applications. From this overview, key considerations and directions for furthering insights and applications in the emergent field of cell viscoelastic mechanotransduction are provided.

**Impact statement:**

Our tissues are viscoelastic: they respond to mechanical stresses and strains in a time-dependent manner. Their mechanical behavior also evolves over time due to growth, aging, remodeling and disease. Understanding cell response to time-dependent and time-evolving mechanical cues is important for a better comprehension of a wide number of pathophysiological processes and for the design of biomimetic substrates, which can be used as physiologically relevant *in vitro* models and in regenerative medicine applications. This review highlights the importance of a more rigorous approach toward viscoelastic material design and testing for cell mechanobiology studies, which embrace the entire spectrum of elasto- and viscotransduction.


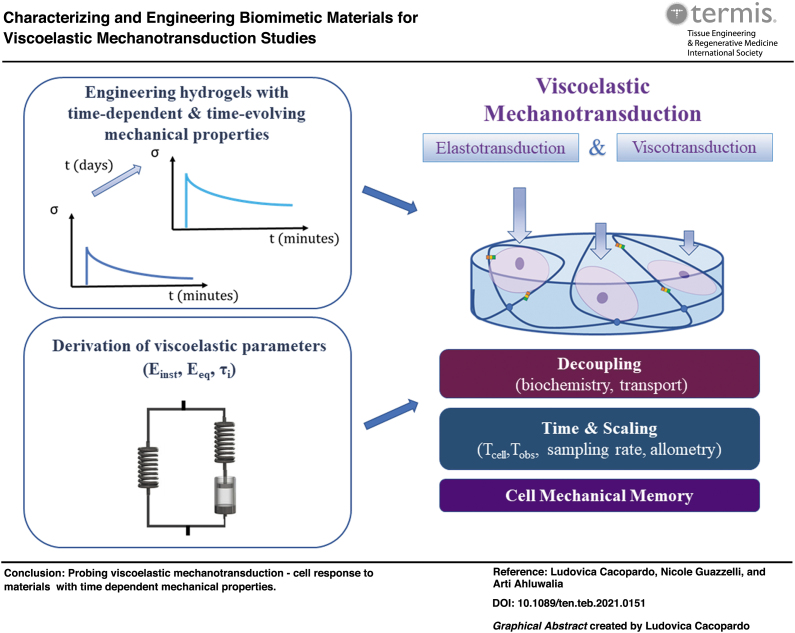


## Introduction

Soft tissues are viscoelastic by nature; their mechanical response is dependent on time and on the rate or frequency of applied stimuli. Moreover, their viscoelasticity also evolves over time because of pathophysiological processes, such as growth, aging, remodeling, repair, and fibrosis. These changes are transmitted to cells, resulting in a two-way flow of interactions between cells and their microenvironment known as dynamic reciprocity.^1–3^

The main structural component of soft tissues is the extracellular matrix (ECM), which is essentially a hydrogel: a biphasic viscoelastic material composed of a solid polymeric network interpenetrating an aqueous solution.^4^ Among the network's components are collagen, responsible for tissue stiffness and strength, and elastin, which endows extensibility and resilience. The behavior of the solid phase can be related to the tissue's elastic properties, usually described by an elastic modulus (E). The interfibrillar liquid is mainly composed of water and solutes such as glycosaminoglycans (GAGs), proteoglycans, and glycoproteins. Its resistance to flow is quantified through viscosity (μ). GAGs and proteoglycans also contribute to tissue compressive stiffness, thanks to their high level of hydration.^2,3,5^

Studying the role of mechanical factors involved in the cell–microenvironment relationship enables the investigation and control of cell behavior through material design. In particular, reproducing the wide range of mechanical behavior manifested by the ECM is fundamental for generating physiologically relevant *in vitro* models and to improve the performance of tissue substitutes for regenerative medicine applications.^6,7^

**FIG. 1.**
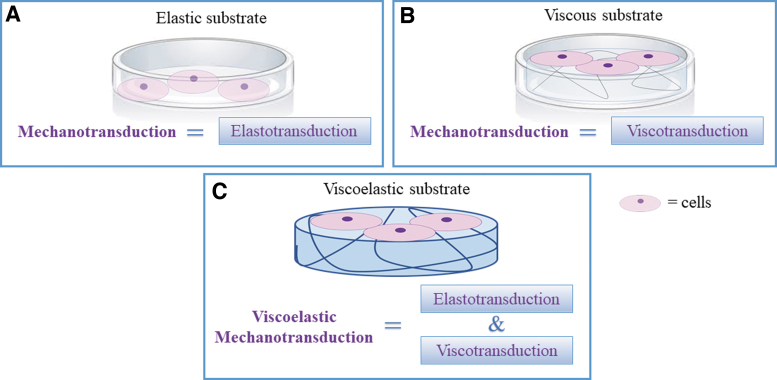
The mechanotransduction spectrum. For elastic **(A)** and viscous **(B)** substrates, mechanotransduction can be, respectively, associated with elastotransduction and viscotransduction. **(C)** For viscoelastic substrates, the two effects cannot be decoupled, and both elastotransduction and viscotransduction may condition cell behavior, hence viscoelastic mechanotransduction. Color images are available online.

Mechanotransduction, the ability of cells to sense, respond and adapt to mechanical signals, has long been investigated as a function of substrate rigidity and is generally associated with cell response to elasticity. But—as tissues are viscoelastic—it is only part of the story. For elastic materials, mechanotransduction can be associated with elastotransduction, i.e., cell response to time invariant stress and strain (Fig. 1 A). In the case of viscous materials (Fig. 1B), we can speak of viscotransduction (cell response to the resistance to flow of a material). Finally, in viscoelastic materials, as the viscous and elastic components cannot be decoupled, cell response to time dependent stress and strain should be referred to as viscoelastic mechanotransduction (Fig. 1C).

The purpose of this review is to provide a reference for scientists wishing to explore the fascinating and emerging field of viscoelastic mechanotransduction by bringing together fundamental aspects of viscoelasticity relevant to the study of soft hydrated biomaterials and tissues, the state of the art in hydrogel design and our current understanding of cell response to time-dependent material behavior.

## Modeling and Measuring Viscoelasticity

Although the theory of viscoelasticity and viscoelastic models are noted in textbooks—and in some excellent reviews^8–15^ —there is some confusion on the terms used to define different models, on the relationships between their viscoelastic constants and the physical interpretation of time and material constants. We begin therefore by introducing some fundamental concepts for understanding and quantifying the mechanical behavior of hydrogels.

For the sake of clarity and consistency, we refer to material mechanical behavior as its intrinsic comportment, which is often described by the terms such as stiff, soft, floppy, and elastic, while the mechanical properties are quantities used to define and describe the behavior through mathematical models. The properties will depend on the nature of the material as well as the models and methods used to extract parameters and quantitative descriptors (time constants, equilibrium and instantaneous elastic moduli or storage and loss moduli).^16^ Additionally, we use the term time dependent when referring to classical viscoelastic behavior in which resistance or compliance to deformation is a function of time, distinguishing it from time-evolving viscoelasticity, which arises from variations in viscoelastic descriptors over time.

### Viscoelastic models

For small deformations, the viscoelastic behavior of hydrogels is related to both solid network deformation and viscous phenomena such as molecular entanglements, chain sliding, and short-range water flow. At higher deformations, water flow occurs over a larger range resulting in “poroviscoelastic” behavior. For a given specimen with characteristic length, L, and a water self-diffusion constant, D, under an experimental observation time, T_obs_, viscoelastic phenomena are observed for characteristic relaxation time τ_relax_ ≈ T_obs_, whereas poroelastic phenomena can be observed when L^2^/D ≈ T_obs_.^17,18^ Given that soft tissues and hydrogels are generally tested under bulk compression or tension, with typical dimensions of the order of 1 cm, poroelastic phenomena will only manifest in the laboratory when experiments last several hours or for high deformations.

**FIG. 2.**
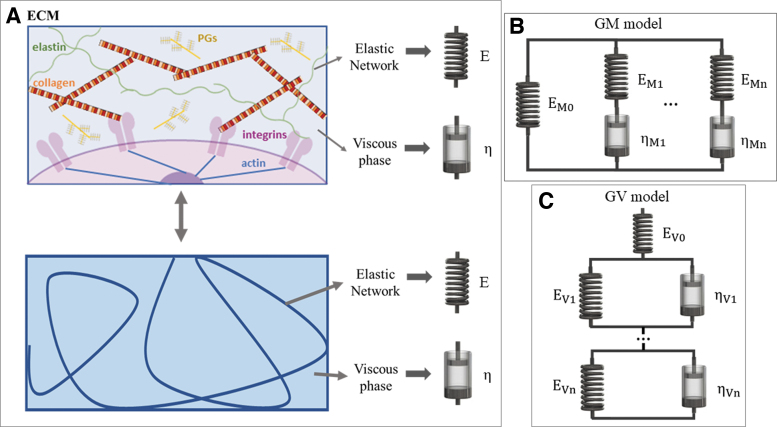
**(A)** Schematic of the ECM and hydrogel analogy with lumped parameters. **(B)** GM model. **(C)** GV model. The subscript n refers to the model order, for example, *n* = 1 is the first order. Alternative nomenclature for these and other models is provided in the Supplementary Information SI2. ECM, extracellular matrix; GM, generalized Maxwell; GV, generalized Voigt. Color images are available online.

Thanks to their general applicability, linear viscoelastic models (also known as lumped parameter models or viscoelastic solid models) are often the preferred choice in biomaterial studies and as such will be the focus of this review. Other mechanical models are summarized in the Supplementary Information SI1. Linear models describe the relationship between deformation (ɛ) and applied stress (σ) through linear combinations of parameters, typically springs and dashpots.^15,19^ The parameters do not have any real physical significance, but it is useful to associate the dashpots (viscous coefficient [η_i_]) with friction, viscosity, sliding, entanglements, and solvent flow and the springs (elastic modulus [E_i_]) with the deformation of the solid network (Fig. 2A). The two elements can be combined to generate different linear viscoelastic models, which can be grouped into two main families: the generalized Maxwell (GM) and generalized Voigt (GV) (Fig. 2B, C). Although they are mechanically equivalent,^15^ the mathematical derivation of the GM model response is simpler for strain inputs, while the GV model response is more suitable for stress inputs. We encourage the use of a common terminology; however, the reader will find that these names are not unique; alternative terms are summarized in the Supplementary Information SI2.

### Viscoelastic testing

Material elastic properties are generally derived by analyzing the stress–strain curve resulting from tensile or compressive tests. The elastic modulus is estimated from the slope of the first linear part of the curve. In the case of viscoelastic materials, this region depends on the strain rate and is referred to as the linear viscoelastic region (LVR). Its slope is defined as the apparent elastic modulus (E_app_).^20,21^ We add here a note about *stiffness*: strictly speaking, stiffness is an extrinsic property, even though it is often used interchangeably with rigidity or elastic modulus. Although the term should be avoided when referring to viscoelastic materials, it is so deeply entrenched in the field of mechanobiology as to be unavoidable. We can relate the stiffness to E_app_ at high strain rates, which in turn closely approximates the instantaneous elastic modulus (E_inst_, see the “Derivation of material descriptors” section).

A variety of testing methods have been developed to derive quantitative descriptors of the time-dependent behavior of viscoelastic materials.^21–23^ They can be classified on the basis of the type of input: a strain or stress step, ramp, or sinusoid (see the Supplementary Information SI3 for details, where we also discuss the difference between bulk and local tests).

### Derivation of material descriptors

For any of the models in Figure 2B, C, equations for the stress or strain as a function of time or frequency can be derived by substituting the Laplace transform of the inputs in the model transfer function (Supplementary Information SI4). The equations can be fitted to the experimental stress–time or strain–time curves or frequency spectra to derive the model lumped parameters (Supplementary Information SI4).

The lumped parameters can be used to derive the material descriptors: E_inst_, the instantaneous modulus, is the initial (i.e., t → 0) elastic response when the viscous components are “shorted” out and do not deform while sustaining the load. E_eq_ is the equilibrium modulus, which represents the response after viscoelastic dynamics have occurred (t → ∞). In these conditions, the piston is completely dissipated, and it cannot support a load; it can be considered analogous to an “open circuit.” The time constants τ_i_ are also descriptors of the material's behavior and are given by the ratio of the coefficients (η_i_/E_i_) for t → τ_i_ for each dashpot and spring pair in the GM or GV models.

**FIG. 3.**
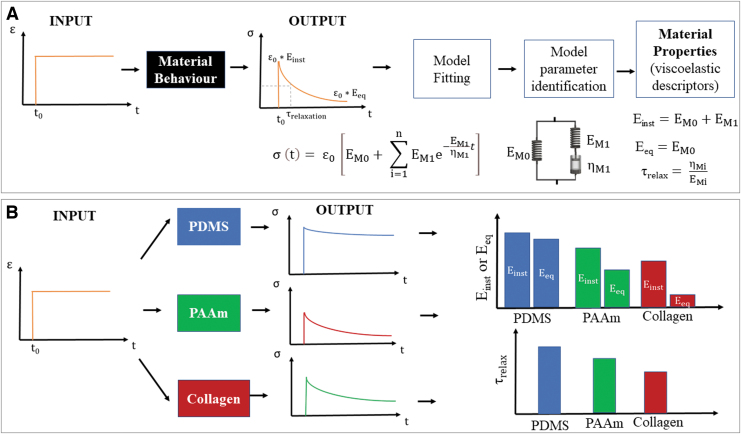
**(A)** Workflow for identifying material viscoelastic properties. **(B)** Mechanical response for different hydrogels: PDMS, PAAM, and soft collagen. For typical observation times, PDMS has solid-like behavior: E_eq_ and E_inst_ are similar and τ_relax_ is higher than T_obs_. Collagen is very soft and liquid-like: E_eq_ and E_inst_ are significantly different and τ_relax_ is low. PAAM generally shows an intermediate viscoelastic behavior. PAAM, polyacrylamide; PDMS, polydimethylsiloxane. Color images are available online.

Figure 3A summarizes the workflow for deriving model parameters and the related viscoelastic descriptors for a strain step input. Examples of typical mechanical responses and descriptors for different hydrogels are shown in Figure 2B. Further theoretical details are illustrated in the Supplementary Information (Supplementary Fig. 2S).

In general—and for the sake of simplicity (see the Supplementary Information SI5)—a single characteristic time, as obtained with the first-order models, is sufficient to describe material viscoelastic behavior. For a strain input, we define a relaxation time (τ_relax_), which is the time in which the stress decays to 1/e (≅ 37%) of the initial stress. For a stress input, the retardation time (τ_retard_) is defined as the time necessary to reach ≅63% (1–1/e) of the equilibrium strain.^24^ The relationship between characteristic relaxation time and retardation time is given in the Supplementary Information SI6.

The descriptors for sinusoidal stimuli are expressed using a complex elastic modulus, E*(ω), composed of a real or storage modulus E′(ω)—related to the ability of the material to return energy—and an imaginary or loss modulus E″(ω)—associated with the energy lost in “internal friction” (e.g., molecular motions, relaxation processes). The ratio between descriptors E″ and E′ defines tan δ, known as the loss or damping factor, which allows the derivation of the relaxation time as the reciprocal of the peak frequency.^25^ δ represents the phase difference between the input and output sinusoid: δ = 0 for ideally elastic materials (all the energy is stored in the material) and δ = 90° for an ideally viscous liquid (all the energy is dissipated).^26^

**FIG. 4.**
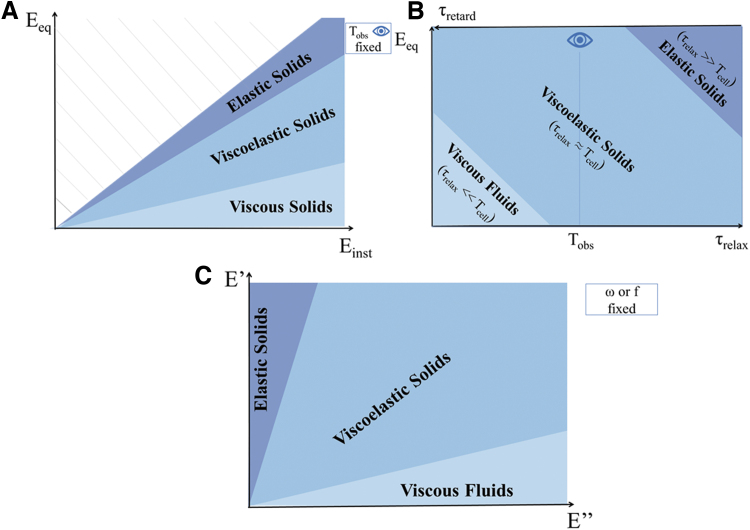
Viscoelastic “phase diagrams” illustrating the elastic to viscous spectrum material of behavior as a function of **(A)** equilibrium and instantaneous modulus (E_eq_ and E_inst_) for a fixed observation time (T_obs_). **(B)** Equilibrium modulus, characteristic relaxation time (τ_relax_), and retardation time (τ_retard_) for a fixed T_obs_. **(C)** Storage and loss modulus (E*′* and E*″*) for a fixed frequency (ω). Color images are available online.

Figure 4 shows the relationships between material behavior and viscoelastic descriptors in the time and frequency domain. When E_inst_ = E_eq_ and τ_relax_ → ∞ (i.e., the stress never relaxes during observation), the material has an ideal elastic or solid-like behavior (Fig. 4A, B). For E_eq_ = 0 and τ_relax_ → 0 (i.e., the stress instantaneously relaxes), the material is defined as a pure viscous or liquid-like (Fig. 4B). However, if τ_retard_→ 0 (i.e., there is no retardation in reaching the equilibrium strain), the material behavior is elastic, *vice versa*, if the retardation time is much longer than the observation time, or → ∞, it is purely viscous. Between these extremes, the material can be considered a viscoelastic solid. Similarly, materials behave as elastic solids for high E′ values and low E″ values and, *vice versa*, they behave as viscous fluids for low E′ and high E″ (Fig. 4C). In all cases, T_obs_ plays a crucial role in determining whether a material can be considered elastic, viscoelastic, or viscous (see section “Time and material behavior”).

Note that the viscoelastic descriptors obtained in the frequency domain can be derived from those in the strain–rate domain and *vice versa*.^21,27,28^ As an example, the equation reporting the relationship between the frequency and time domain descriptors for the first-order GM model can be found in the Supplementary Information SI4.

### Time and material behavior

That the perception of viscoelasticity depends on the observation time was noted by Reiner, who defined the Deborah number as the ratio between the relaxation time and observation time: De = τ_relax_/T_obs_.^29^ In the case of De>>1, the material is expected to manifest elastic solid behavior; while, if De<<1, the material will behave as a viscous liquid. When the relaxation time and observation time are comparable, the material shows both “fluid-like” and “solid-like” characteristics and is defined as viscoelastic^29,30^ (Fig. 4B). To meaningfully compare Deborah numbers, either the characteristic times or the observation time should be fixed. In general, the observation time is easier to control in an experiment. However, comparing results across different studies can be a challenge since both experimental times and material relaxation times may vary.

Material viscous behavior can also be related to the liquid phase viscosity (μ), which quantifies liquid resistance to flow and depends on the concentration and the molecular weight of solutes^19^ or of un-cross-linked polymers.^31^ Liquid phases with a low μ or high fluidity (1/μ) are associated with a liquid-like behavior, whereas viscous solutions with high μ exhibit solid-like behavior. Quite often, this concept causes confusion as one (erroneously) tends to associate (high) viscosity with liquid behavior.

Polymer chain entanglements and interactions between the polymeric chains and the liquid phase also modulate μ. Strong interactions are likely to be associated with “bound water,” which does not flow easily through the polymeric network, whereas weaker interactions allow higher water mobility.^19,32^ Table 1 summarizes these concepts.

**Table 1.** Properties and Phenomena Associated with Material Elastic and Viscous Behavior

	Elastic behavior	Viscous behavior
Also defined as:	“Solid-like”	“Liquid-like”
	Conservative	Dissipative
	Time independent	Time dependent
	Materials with memory	Materials with no memory
Associated with		
Polymer concentration/cross-linking	↑	↓
Polymeric–liquid interaction	↑	↓
Polymer chain entanglements	↓	↑
Viscosity, elastic, or shear moduli and characteristic times		
Liquid phase resistance to “flow,” μ	↑	↓
Liquid phase 1/μ (fluidity)	↓	↑
E′ or G′	↑	↓
E″ or G″	**↓**	↑
δ	↓	↑
τ_relaxation_ or De	↑	↓
τ_retardation_	↓	↑
Strain rate dependence	↓	↑

G′ is the storage shear modulus and G″ is the loss shear modulus.

↑, increase; ↓, decrease.

## Biological Viscoelasticity

### Time-evolving viscoelasticity

How do tissue mechanics change over the different stages of life and in health and disease? During growth, there is a high turnover of ECM components, which contributes to tissue remodeling and reinforcement in response to the mechanical stimuli.^33,34^ In adulthood, feedback mechanisms based on ECM reorganization and cross-linking and a precise balance between degradation and deposition^2,3^ guarantee homeostasis and adaptation.

During maturation and remodeling (e.g., wound healing), collagen and elastin undergo precisely modulated cross-linking through the action of enzymes. The resulting increase in stiffness helps tissues to achieve structural resistance.^2,35,36^ Enzymatic mediated cross-linking reaches a plateau during maturation, but tissue rigidity continues to increase with age. ECM aging is characterized by cross-linking arising from uncontrolled nonenzymatic oxidative reactions with glucose (glycation). Although these reactions are slower than enzymatic ones, the relatively long half-lives of ECM components (≈10 years for collagen, ≈60 years for elastin) make glycation-mediated cross-linking significant.^2,35,36^ Moreover, the deceleration of collagen turnover coupled with the extremely low elastin renewal rate implies irreversible changes in tissue form and function, which result from mechanical fatigue, damage or proteolytic degradation. Since ECM viscosity is directly proportional to the concentration of its constituents, the loss of collagen results in an increase in fluid mobility in tissues, which is manifested by a decrease in viscosity.^2,37–39^

However, excessive or uncontrolled collagen deposition and cross-linking are responsible for organ dysfunction in fibrotic and degenerative diseases (e.g., pulmonary fibrosis, liver cirrhosis, cardiovascular disease and systemic sclerosis). These conditions are associated with fibroblast hyperproliferation, in which the cells tend to differentiate into collagen-secreting myofibroblasts, and with the upregulation of cross-linking enzymes such as lysyl oxidase.^3,40^ Table 2 recaps the main factors involved in different pathophysiological process and consequent effects on tissue mechanical behavior. Elasticity and viscosity trends have been considered separately to highlight their contributions. However, tissue elastic and viscous behaviors are intrinsically related and, typically, cannot be decoupled.

**Table 2.** Tissue Mechanical Variations During Pathophysiological Processes

	Growth/maturation	Adulthood	Aging	Fibrosis
E (elastic modulus/behavior)	↑	∼	↑	↑
μ (viscosity)	↑	∼	↓	↑
η (viscous coefficient/behavior)	↓	∼	Depends on the relative variation of E and μ	↓
Main factors involved	Enzymatic controlled cross-linking Collagen physiological remodeling with loads ECM deposition	ECM reorganization Balance between ECM deposition and turnover	Glycation Collagen depletion Elastin damage accumulation	LOX upregulation Collagen hyperproduction
Time scales	From days to years	From days to years	Years	Years
References	2,33–36,89,90	2,3	2,37–39,56,91–94	2,94,95

As tissues mature, age, or remodel over time, the mechanical properties also evolve.

∼, almost constant; ECM, extracellular matrix; LOX, lysyl oxidase.

### Understanding cell response to viscoelasticity

Since Pelham and Wang first observed the effect of substrate elasticity on fibroblasts in 1997, mechanobiology studies have increased exponentially.^41^ Historically, cells have been cultured on rigid plasticware, and controlling and measuring material elastic constants are relatively easy. As a consequence, cell response to viscoelasticity is still poorly understood and results are often contradictory. Some studies report on an increase of cell spreading area and differentiation on viscoelastic substrates with respect to elastic ones,^42–46^ whereas others describe the opposite.^44,47–49^ These conflicting results have been related to the different cell types and culture conditions (e.g., two-dimensional [2D] vs. three-dimensional [3D]).^44,50–52^ Cell spreading depends on cytoskeletal tension, which in turn depends on substrate mechanics. Thus, one might expect that substrate forces relax over time on soft time-dependent materials, resulting in lower cytoskeletal tension and spreading. However, in some cases, the higher deformability of these substrates may give rise to localized ligand clustering resulting in local stiffening in response to cell movements such that spreading is improved with respect to an elastic substrate with the same ligand density.^42,53^ Moreover, in 3D, cell migration is likely easier in soft gels than in more elastic ones; since the cells are not “caged” in a stiff matrix, they can move and produce their own extracellular environment.^50,51,53^ Cell volumetric expansion, which in 3D is determined by the “dimensionality” of cell-ECM interaction, is indeed an important factor able to drive cell spreading and commitment. For instance, Caliari *et al.* reported that mesenchymal stem cells (MSCs) showed increased spreading and YAP/TAZ nuclear translocation in 2D while the opposite was observed in 3D, suggesting that cell behavior is not only regulated by stiffness but also by the complex interplay of environmental factors.^54^ Major *et al.* found similar results with adipose-derived MSCs. They also showed that fast relaxing gels allowed greater cell volume expansion with respect to slow relaxing ones.^55^

The main studies on cell response to viscoelasticity are summarized in Table 3, highlighting cell types and their behavior as a function of substrate viscoelasticity.

**Table 3.** Summary of the Main Studies on Cell Response to Viscoelasticity

Cell response with increasing gel viscous behavior	Hydrogel type		Cell type	Reference
↑ Spreading, proliferation, differentiation	2D	PAAm	MSCs	43
		PAAm	Huh7 cell line, hepatocellular carcinoma cells	52
		PEG thioester norbornene	3T3 fibroblasts	45
		RGD alginate	3T3 fibroblasts	57
	3D	Hyaluronic acid	MSCs	54
		PEG	C2C12 myoblasts	96
		RGD PEG alginate	MSCs, 3T3 fibroblasts	42,58
		Hyaluronic acid and collagen	MSCs	58
↓ Spreading, proliferation, differentiation	2D	PAAm	3T3 fibroblasts	47
		PAAm	Hepatocytes	52
		PAAm loaded with carbonyl iron particles	MSCs	46
		Collagen loaded with carbonyl iron particles	Coronary artery smooth muscle cells	62
		Hyaluronic acid	MSCs	54,61

2D, two-dimensional; 3D, three-dimensional; MSCs, mesenchymal stem cells; PAAm, polyacrylamide; PEG, polyethylene glycol; RGD, arginine, glycine, aspartate.

## Engineering Hydrogel Viscoelasticity

To recap, cells and tissues are exquisitely mechanoresponsive at all stages of life, in sickness and in health. Reproducing the stiffness and time-dependent as well as time-evolving range of mechanical behavior manifested by the ECM is fundamental for better understanding cell–material interaction and designing materials for biomedical applications.^6,51^

### Modulation of viscoelastic (time-dependent) properties

Hydrogel viscoelasticity can be modulated using different techniques, and the gel will have (approximately) constant mechanical properties throughout the culture period. An increase in the polymer or cross-linker concentration (and consequently in the degree of cross-linking of the polymer network) results in a shift toward a more elastic behavior.^23,43,47,56^ For instance, by varying the proportion of acrylamide and bis-acrylamide, Cameron *et al.* demonstrated that MSC spreading and differentiation increased with increasing loss moduli (corresponding to decreasing gel relaxation times).^43^ Similarly, using polymers with different molecular weight or modulating the formation of reversible cross-links results in gels with different viscoelastic behavior.^49,57^ Alternatively, the mobility of the polymeric chains can be improved by the addition of spacers, thus increasing gel dissipative behavior.^49,57^ The use of alginate with different molecular weights and of alginate with polyethylene glycol (PEG) spacers allow greater spreading in both MSCs and fibroblasts.^42,58^ As these methods mainly act on the gel polymeric network, they concomitantly alter the elastic and the viscous component since the two phenomena cannot be decoupled.^47,49^ Cacopardo *et al.* reported an alternative and unique approach whereby the gel's viscous properties—and hence τ—are directly controlled by tuning the liquid phase viscosity.^19^

### Modulation of time-evolving properties

A few recent efforts to mimic tissue dynamics and study how cells adapt and respond to a mechanically evolving context have been reported. Generally, the viscoelastic properties of gels can be modulated over time through agents, which modulate the formation or degradation of cross-links, although not all methods can be used in the presence of cells. For example, enzymatic cross-linkers exploit enzyme reaction kinetics to enable the tuning of gel viscoelastic properties over time.^40,56^ Chemical reactions with slow kinetics can also be used to generate gels with time-evolving viscoelasticity.^45,59–61^ Finally, the use of responsive materials represents an intriguing strategy to modulate gel viscoelasticity on-demand.^46,62^ As an example, MSC spreading on magneto-responsive gels was reduced when the gels switched from elastic to liquid-like behavior.^46^ Table 4 summarizes some of the approaches used to modulate the viscoelastic behavior of biocompatible hydrogels. Notably, only two studies so far have investigated time-evolving hydrogels. More efforts in this direction are thus needed to engineer mechano-mimetic models able to replicate pathophysiological processes *in vitro*.

**Table 4.** Summary of Cross-linking Strategies to Modulate Hydrogel Viscoelasticity

	Method	Material	Effect	Compatibility with 3D cell encapsulation	Direct modulation of the viscous phase	Reference
Viscoelastic substrates (time-dependent behavior, constant viscoelastic descriptors)	Polymer concentration, cross-linker ratio	PEG-alginate	↑ [PEG spacer]: ↑ G′, ↓ G″	Yes	No	57
		PAAm	↑ [bis-acrylamide]: ↑ G′, ↓G″	No	No	43
		HMWLP-PAAm	↑[HMWLP]: ∼ G′, ↑ G″	No	No	47
		Boronate ester-PEG	↑ [boranate]: ∼ G′, ↑ G″	Not specified	No	49
	Liquid phase viscosity	Dextran – Agarose/PAAm	↑ [dextran]: ∼ E_eq_, ↓ E_inst_, ↓ τ_relax_	Yes	Yes	19
	Chemical cross-linking	GTA-Gelatin	↑ [GTA]: ↑ E_eq_, ↑ E_inst_, ↑ τ_relax_	No	No	23,56
Time-evolving viscoelastic substrates (time-evolving behavior, viscoelastic descriptors are a function of time)	Enzymatic or chemical reactions with slow kinetics	mTG-GTA-Gelatin	↑ incubation time: ↑ E_eq_, ↑ E_inst_, ↑ τ_relax_ (after 1 day)	No	No	56
		mTG-Gelatin	↑ incubation time: ↑ E_eq_, ↑ E_inst_, ↓τ_retard_	Yes	No	40
		MA-HA	↑ incubation time: ↑ G′, ↑ G″	Yes	No	59
	Photo-cross-linking (e.g., UV)	MA-HA	↑ exposure time: ↑ G′, ↑ G″	Yes	No	60
		NA-HA	↑ exposure time: ∼ G′, ↓ G″	Yes	No	61
		Thioester-PEG	↑ exposure time: ↓ relaxation curve slope	Yes	No	45
	Magnetic	Fe(CO)_5_ – PAAm/Collagen	↑ exposure time: ↑ G′, ↑ G″	Not specified	No	46,62

GTA, glutaraldehyde; HA, hyaluronic acid; HMWLP, high-molecular-weight linear polymers; MA, methacrylate; mTG, microbial transglutaminase; NA, nitrobenzyl acrylate; UV, ultraviolet.

## Perspectives for Viscoelastic Mechanotransduction Studies

As we explore the new field of cell viscoelastic mechanotransduction through viscoelastic material engineering, it is important to bear in mind a number of factors, which are crucial for posing specific research questions and interpreting data. They will also impact on the way in which experiments are designed.

### Mechanosensing time and spatial scales

Cells are able to sense substrate mechanics, thanks to the formation of focal adhesions (FAs), which are molecular complexes that act like bridges between the ECM and the cytoskeleton. Typically, the size of FAs is between 0.25 and 10 μm and their lifetime is in the range of tens of minutes, depending on cell type and substrate. The cell integrates these local signals eliciting a measurable response.^63–66^

The ambiguity of the results on cell response to viscoelasticity is not only due to different experimental conditions (such as cell types, substrate materials, mechanical testing methods) but also due to an intrinsic difficulty in their interpretation due to the diverse spatial and time scales involved. As discussed in the Supplementary Information SI3, the mechanical testing scales range from nanometers to centimeters and should be selected according to the material under testing.^67^ Moreover, the ability of the cells to sense and respond to the time-dependent substrate properties should be considered in the light of the range of time scales of the different players involved. The first player is the substrate, which has one or more characteristic relaxation times. The second player is the cell, whose response is related to its ability to bind to adhesion molecules and transduce the substrate tension into a biochemical signal.

According to the molecular clutch theory, cell adhesion is related to both the time needed to form cell–ECM bonds (t_binding_) and their lifetime (t_lifetime_)^52^; this interval of time can be referred as the cell sensing time (T_cell_). Typically, t_lifetime_ is higher than t_binding_, allowing the formation of stable bonds able to trigger mechanotransduction pathways. As shown in Figure 6A, the substrate characteristic relaxation times (τ_relax_) should be comprised within the cells’ sensing time window to allow them to “perceive” the substrate dynamics and activate pathways, which may result in a quantifiable difference in their behavior in response to substrate viscoelasticity. If T_cell_ is too short with respect to τ_relax_, the cells can sense only the instantaneous material response. However, if the time required to form (and maintain) the bonds exceeds τ_relax_, cells are likely to “perceive” only the equilibrium material response.^68,69^ As an example, using 3T3 fibroblasts seeded on hyaluronic acid gels, Gong *et al.* showed that substrate viscoelasticity can regulate cell spreading depending on the relationship between substrate relaxation time and cell binding time and duration.^68^ Therefore, the investigation of cell response to viscoelasticity cannot prescind from accurate design of substrate viscoelastic dynamics according to the cell sensing time window, which may differ according to cell type or disease states.^44^

The third factor is the observation time (T_obs_)—which includes intermediate and end measuring points—compared with the characteristic time of the biological process under investigation. Frequent interrogation of slow processes would constitute oversampling, whereas undersampling of fast ones may give rise to aliasing. Moreover, T_obs_ should be longer than both characteristic viscoelastic times and biological times of interest to capture a complete picture of the processes and their interdependencies. As T_obs_ and sampling frequency depend on the mechanical testing method (Supplementary Information SI3), experimental parameters should be carefully chosen to obtain meaningful results.

### Time and mechanical scaling

The general allometric equation is *Y = aM^b^*, where *Y* is the physiological parameter of interest (metabolic rate, cell number, etc.), *M* is the body mass, *b* is the allometric scaling exponent, and *a* is a constant. “Physiological time,” defined as the species-dependent chronological time period required to complete a physiological event, is known to vary between different sized organisms following allometric scaling laws. The scaling exponent for physiological times (gestation time, lifespan, etc.) is ubiquitously *b ≈ 1/4*.^70^ Thus, characteristic times and time-evolving processes in downscaled *in vitro* cultures are expected to be shorter than *in vivo*. In fact, time scaling in cell cultures is a well-known phenomenon,^71,72^ although its extrapolation and translation to whole organisms have never been formalized as has the scaling of metabolic rate.^73,74^

Mechanical tailoring *in vitro* is typically focused on mimicking the amplitude of the mechanical—be they elastic or viscoelastic—properties associated with biological processes, often neglecting to consider their typical timescales. In this perspective, it would be of interest to investigate the relationships between the timescales observed in *in vitro* mechanotransduction experiments and their *in vivo* parallels to tease out scaling laws for cellular processes associated with mechanical cues.

### Mechanical memory

Recent studies demonstrate that cells remember the past mechanical characteristics of their environment and their ability to respond to new mechanical stimuli depends on their mechanical history. For example, stem cell priming on stiff substrates reduces their epigenetic plasticity (i.e., the ability to modify their phenotype), blocking the transcription of new genes required for the adaptation to a new environment.^75,76^ This type of memory has also been observed for other cells such as lung fibroblasts, which showed persistent myofibroblast activity after 3 weeks on polydimethylsiloxane substrates with a pathological stiffness, even after switching to a softer substrate.^77^

The longer cells are cultured on stiff substrates, the less they respond to substrates with different mechanical properties (Fig. 6C). In particular conditions, depending on the combination of substrate stiffness and culture duration, cell mechanical memory can be restored.^75,78^ The promoter of this reversible memory effect is thought to be the YAP/TAZ complex, which acts as a mechanical rheostat mediating the mechanical dosing. Yang *et al.* were the first to demonstrate that YAP/TAZ translocation in MSCs was reversible after 7 days of culture of stiff PEG gels.^78^

Nonetheless, the routine use of plasticware typical of standard cell extraction and culture protocols is likely to affect the cell behavior observed during experiments.^78,79^ Researchers should be aware of these issues when investigating mechanobiological responses, and we suggest that efforts be made to establish new standardized cell culture protocols for preserving or resetting native cell mechanosensitivity.

### Decoupling mechanics from other factors

**FIG. 5.**
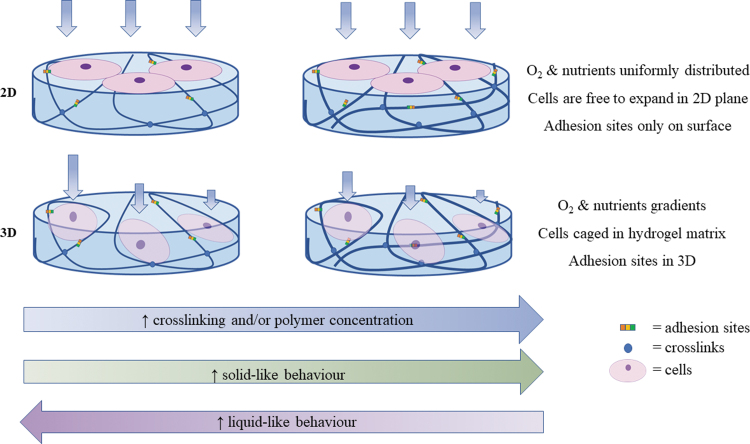
Difference between 2D and 3D gels in mechanobiology studies: when altering gel mechanical behavior in 3D (typically increasing polymer concentration or cross-linking), properties such as biochemical cues (adhesion sites), transport (oxygen and nutrient diffusion), and dimensionality (volume available for cell expansion) also differ. The interacting effects may cloud the understanding of mechanical response in 3D. 2D, two-dimensional; 3D, three-dimensional. Color images are available online.

When designing microenvironments for mechanobiology studies, in addition to mechanical factors, it is important to provide the cells with adequate topographic and biochemical cues, and, especially in 3D, to guarantee sufficient space for cell expansion. However, little attention is paid to the consideration of these interacting factors, which may lead to misinterpretation of results. In fact, typical experiments consist of comparing cell behavior on 2D plastic substrates with that of cells on 2D or 3D gels. Not only are the mechanical properties different, but features such as surface roughness, surface chemistry, and haptotactic cues may also differ^80,81^ (Fig. 5).

**FIG. 6.**
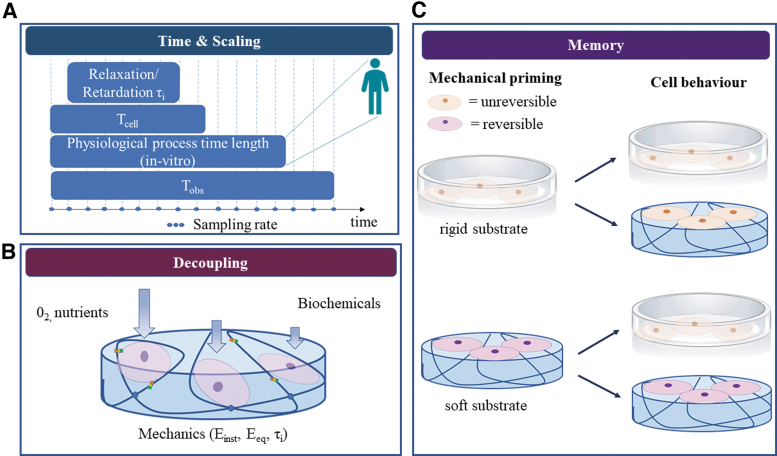
Future challenges in the engineering and design of biomimetic viscoelastic materials: **(A)** experimental timing (observation time and sampling rate) should be set according to the (*in vitro*) duration of the physiological process under study, which ideally should be rescaled with respect to *in vivo* times following allometric laws. **(B)** Cell response to substrate mechanics should be decoupled from their responses to biochemical and transport cues. **(C)** The mechanical history of cells should be considered so as to meaningfully interpret their response on different substrates. Cells may not be able to respond to soft, viscoelastic substrates if they undergo irreversible mechanical priming on rigid substrates (*top*). However, if primed on soft substrates able to mimic the native cell environment, cells may retain their ability to respond to changes in substrate mechanics (*bottom*). Color images are available online.

There is a considerable body of work focusing on the decoupling of stiffness from ligand density^82–84^ and some articles dealing with stiffness and topography^85^ or mineral content.^86^ The issue of decoupling interacting effects in mechanotransduction studies is exacerbated in 3D gels, wherein the increase of polymer concentration and cross-linking not only alters the mechanical behavior of gels but also affects oxygen, nutrient diffusion, and cell volume.^87^ As a consequence, changes in cell response may be influenced, or even overshadowed by factors other than the mechanical properties of the cell environment.^88^ A current challenge for *in vitro* research is the isolation or decoupling of mechanical properties from other variables to fully understand and thus direct cell behavior by specifically tuning environmental cues, which engineers can design and control (Fig. 6B).

## Conclusions

This review aims at encouraging a more rigorous and harmonized approach toward investigations in mechanotransduction, embracing the entire spectrum of elasto- and viscotransduction to include viscoelastic mechanotransduction. It highlights the importance of designing biomimetic viscoelastic materials with characteristic time responses, which are compatible with experimental times and also relevant to physiological/pathological times under investigation. Some of the future challenges in the field of viscoelastic mechanotransduction (summarized in Fig. 6) are discussed. Among these, there are novel strategies to control factors, which influence cell response, decoupling mechanical properties from substrate biochemistry, topographical features, and mass transport.

An intriguing question, as yet unexplored, is that of time scaling of cellular responses from *in vitro* to *in vivo* and *vice versa*. A final challenge is the definition of cell culture protocols, which can reset cell mechanical memory to a common baseline, crucial for facilitating meaningful comparisons across different studies.

## Supplementary Material

Supplemental data

## References

[1] Ingber, D.E. Tensegrity: the architectural basis of cellular mechanotransduction. Annu Rev Physiol 59, 575, 1997. 9074778 10.1146/annurev.physiol.59.1.575

[2] Cox, T.R., and Erler, J.T. Remodeling and homeostasis of the extracellular matrix: implications for fibrotic diseases and cancer. Dis Model Mech 4, 165, 2011. 21324931 10.1242/dmm.004077 PMC3046088

[3] Humphrey, J.D., Dufresne, E.R., and Schwartz, M.A. Mechanotransduction and extracellular matrix homeostasis. Nat Rev Mol Cell Biol 15, 802, 2014. 25355505 10.1038/nrm3896 PMC4513363

[4] Selfe, J. Fundamentals of biomechanics. Physiology 86, 163, 2000.

[5] Dunn, M.G., and Silver, F.H. Viscoelastic behavior of human connective tissues: relative contribution of viscous and elastic components. Connect Tissue Res 12, 59, 1983. 6671383 10.3109/03008208309005612

[6] Stowers, R.S., Allen, S.C., and Suggs, L.J. Dynamic phototuning of 3D hydrogel stiffness. Proc Natl Acad Sci 112, 1953, 2015. 25646417 10.1073/pnas.1421897112 PMC4343123

[7] Mastrorocco, A., Cacopardo, L., Martino, N.A., *et al.* One-step automated bioprinting-based method for cumulus-oocyte complex microencapsulation for 3D in vitro maturation. PLoS One 15, 1, 2020. 10.1371/journal.pone.0238812 PMC7485809 32915922

[8] Ebenstein, D.M., and Pruitt, L.A. Nanoindentation of soft hydrated materials for application to vascular tissues. J Biomed Mater Res 69A, 222, 2004. 10.1002/jbm.a.20096 15057995

[9] Buffinton, C.M., Tong, K.J., Blaho, R.A., Buffinton, E.M., and Ebenstein, D.M. Comparison of mechanical testing methods for biomaterials: Pipette aspiration, nanoindentation, and macroscale testing. J Mech Behav Biomed Mater 51, 367, 2015. 26295450 10.1016/j.jmbbm.2015.07.022

[10] Rettler, E., Hoeppener, S., Sigusch, B.W., and Schubert, U.S. Mapping the mechanical properties of biomaterials on different length scales: depth-sensing indentation and AFM based nanoindentation. J Mater Chem B 1, 2789, 2013. 32260867 10.1039/c3tb20120a

[11] Attard, P. Measurement and interpretation of elastic and viscoelastic properties with the atomic force microscope. J Phys Condens Matter 19, 473201, 2007.

[12] Cohen, S.R., and Kalfon-Cohen, E. Dynamic nanoindentation by instrumented nanoindentation and force microscopy: a comparative review. Beilstein J Nanotechnol 4, 815, 2013. 24367751 10.3762/bjnano.4.93 PMC3869246

[13] Chen, D.L., Yang, P.F., and Lai, Y.S. A review of three-dimensional viscoelastic models with an application to viscoelasticity characterization using nanoindentation. Microelectron Reliab Elsevier Ltd 52, 541, 2012.

[14] Gibson, R.F. A review of recent research on nanoindentation of polymer composites and their constituents. Compos Sci Technol 105, 51, 2014.

[15] Serra-Aguila, A., Puigoriol-Forcada, J.M., Reyes, G., and Menacho, J. Viscoelastic models revisited: characteristics and interconversion formulas for generalized Kelvin–Voigt and Maxwell models. Acta Mech Sin Xuebao 35, 1191, 2019.

[16] Mattei, G., and Ahluwalia, A. Sample, testing and analysis variables affecting liver mechanical properties: a review. Acta Biomater 45, 60, 2016. 27596489 10.1016/j.actbio.2016.08.055

[17] Malandrino, A., and Moeendarbary, E. Poroelasticity of living tissues [Internet]. Encycl Biomed Eng 2017. 10.1016/B978-0-12-801238-3.99932-X

[18] Hu, Y., and Suo, Z. Viscoelasticity and poroelasticity in elastomeric gels. Acta Mech Solida Sin 25, 441, 2012.

[19] Cacopardo, L., Guazzelli, N., Nossa, R., Mattei, G., and Ahluwalia, A. Engineering hydrogel viscoelasticity. J Mech Behav Biomed Mater 89, 162, 2019. 30286375 10.1016/j.jmbbm.2018.09.031

[20] Collinsworth, A.M., Zhang, S., Kraus, W.E., and Truskey, G.A. Apparent elastic modulus and hysteresis of skeletal muscle cells throughout differentiation. Am J Physiol Cell Physiol 283, 1219, 2002. 10.1152/ajpcell.00502.2001 12225985

[21] Cacopardo, L., Mattei, G., and Ahluwalia, A. A new load-controlled testing method for viscoelastic characterisation through stress-rate measurements. Materialia 9, 100552, 2020.

[22] Tirella, A., Mattei, G., and Ahluwalia, A. Strain rate viscoelastic analysis of soft and highly hydrated biomaterials. J Biomed Mater Res Part A 102A, 3352, 2014. 10.1002/jbm.a.34914 PMC4304325 23946054

[23] Mattei, G., Cacopardo, L., and Ahluwalia, A. Micro-mechanical viscoelastic properties of crosslinked hydrogels using the nano-Epsilon dot method. Materials 10, 889, 2017. 10.3390/ma10080889 PMC5578255 28767075

[24] Worgull, M. Chapter 3- Molding materials for hot embossing. In: Worgull, M.B.T.-H.E., ed. Micro and Nano Technology. Boston, MA: William Andrew Publishing, 2009.; pp. 57–112.

[25] Menard, K.P. Dynamic Mechanical Analysis: A Practical Introduction. Second ed. Oxfordshire, United Kingdom: Taylor & Francis Group, 2008.

[26] Vodovotz, Y., Hallberg, L., and Chinachoti’, P. Effect of aging and drying on thermomechanical properties of white bread as characterized by dynamic mechanical analysis (DMA) and differential scanning calorimetry (DSC). Cereal Chem 73, 264, 1996.

[27] Bartolini, L., Iannuzzi, D., and Mattei, G. Comparison of frequency and strain-rate domain mechanical characterization. Sci Rep 8, 13697, 2018. 30209311 10.1038/s41598-018-31737-3 PMC6135832

[28] Wang, L., and Liu, X. The comparison of measurement of mechanical properties of viscoelastic material by dynamic and creep nanoindentation with spherical tip. International Conference on Manipulation, Manufacturing and Measurement on the Nanoscale, IEEE, Taipei, Taiwan, 2014.

[29] Reiner, M. The Deborah number. Phys Today 17, 62, 1964.

[30] Poole, R.J. The Deborah and Weissenberg numbers. Br Soc Rheol Rheol Bull 53, 32, 2012.

[31] Debye, P. The intrinsic viscosity of dilute polymer solutions. J Chem Phys 14, 636, 1946.

[32] Wu, S., and Shanks, R.A. Conformation of polyacrylamide in aqueous solution with interactive additives and cosolvents. J Appl Polym Sci 89, 3122, 2003.

[33] Shraiman, B.I., Wieschaus, E.F., Mar, N., and Shraimant, B. Mechanical feedback as a possible regulator of tissue growth. Proc Natl Acad Sci 102, 3318, 2016. 10.1073/pnas.0404782102 PMC552900 15728365

[34] Ingber, D.E. Mechanical control of tissue growth: function follows form. Proc Natl Acad Sci 102, 11571, 2005. 16091458 10.1073/pnas.0505939102 PMC1188017

[35] Bailey, A.J. Molecular mechanisms of ageing in connective tissues. Mech Ageing Dev 122, 735, 2001. 11322995 10.1016/s0047-6374(01)00225-1

[36] Snedeker, J.G., and Gautieri, A. The role of collagen crosslinks in ageing and diabetes—The good, the bad, and the ugly. Muscles Ligaments Tendons J 4, 303, 2014. 25489547 PMC4241420

[37] Karathanasopoulos, N., and Ganghoffer, J. Investigating the effect of aging on the viscosity of tendon fascicles and fibers. Front Bioeng Biotechnol 7, 1, 2019. 31157219 10.3389/fbioe.2019.00107 PMC6529838

[38] Sobolewsky, E., Ryan, E., Thompson, B., and Conchola, E. The influence of age on the viscoelastric stretch response. J Strength Cond Res 28, 1106, 2014. 24276306 10.1519/JSC.0000000000000326

[39] Dierick, F., Detrembleur, C., Trintignac, G., and Masquelier, E. Nature of passive musculoarticular stiffness increase of ankle in female subjects with fibromyalgia syndrome. Eur J Appl Physiol 111, 2163, 2011. 21298443 10.1007/s00421-011-1850-2

[40] Cacopardo, L., and Ahluwalia, A. Engineering and monitoring 3D cell constructs with time-evolving viscoelasticity for the study of liver fibrosis in vitro. Bioengineering 8, 106, 2021. 34436109 10.3390/bioengineering8080106 PMC8389340

[41] Pelham, R., and Wang, Y. Cell locomotion and focal adhesions are regulated by substrate flexibility. Proc Natl Acad Sci 94, 13661, 1997. 9391082 10.1073/pnas.94.25.13661 PMC28362

[42] Chaudhuri, O., Gu, L., Klumpers, D., *et al.* Hydrogels with tunable stress relaxation regulate stem cell fate and activity. Nat Mater 15, 326, 2016. 26618884 10.1038/nmat4489 PMC4767627

[43] Cameron, A.R., Frith, J.E., and Cooper-White, J.J. The influence of substrate creep on mesenchymal stem cell behaviour and phenotype. Biomaterials 32, 5979, 2011. 21621838 10.1016/j.biomaterials.2011.04.003

[44] Mandal, K., Gong, Z., Rylander, A., Shenoy, V., and Janmey, P. Opposite responses of normal hepatocytes and hepatocellular carcinoma cells to substrate viscoelasticity†. Biomater Sci 8, 1316, 2013. 10.1039/c9bm01339c PMC8317444 31903466

[45] Carberry, B.J., Rao V.V., and Anseth, K.S. Phototunable viscoelasticity in hydrogels through thioester exchange. Ann Biomed Eng 48, 2053, 2020. 32020346 10.1007/s10439-020-02460-w PMC7334082

[46] Abdeen, A.A., Lee, J., Bharadwaj, N.A., Ewoldt, R.H., and Kilian, K.A. Temporal modulation of stem cell activity using magnetoactive hydrogels. Adv Healthc Mater 5, 2536, 2016. 27276521 10.1002/adhm.201600349 PMC5061612

[47] Charrier, E.E., Pogoda, K., Wells, R.G., and Janmey, P.A. Control of cell morphology and differentiation by substrates with independently tunable elasticity and viscous dissipation. Nat Commun 9, 1, 2018. 29386514 10.1038/s41467-018-02906-9 PMC5792430

[48] Shirke, P.U., Goswami, H., Kumar, V., *et al.* “Viscotaxis”—directed migration of mesenchymal stem cells in response to loss modulus gradient. Acta Biomater S1742-7061, 00572, 2021. 10.1016/j.actbio.2021.08.039 PMC7616456 34469788

[49] Marozas, I.A., Anseth, K.S., and Cooper-White, J.J. Adaptable boronate ester hydrogels with tunable viscoelastic spectra to probe timescale dependent mechanotransduction. Biomaterials 223, 119430, 2019. 31493696 10.1016/j.biomaterials.2019.119430 PMC6764851

[50] Chaudhuri, O. Viscoelastic hydrogels for 3D cell culture. Biomater Sci 5, 1480, 2017. 28584885 10.1039/c7bm00261k

[51] Chaudhuri, O., Cooper-White, J., Janmey, P.A., Mooney, D.J., and Shenoy, V.B. Effects of extracellular matrix viscoelasticity on cellular behaviour. Nature 584, 535, 2020. 32848221 10.1038/s41586-020-2612-2 PMC7676152

[52] Mandal, K., Gong, Z., Rylander, A., Shenoy, V.B., and Janmey, P.A. Opposite responses of normal hepatocytes and hepatocellular carcinoma cells to substrate viscoelasticity. Biomater Sci 8, 1316, 2020. 31903466 10.1039/c9bm01339c PMC8317444

[53] Cantini, M., Donnelly, H., Dalby, M.J., and Salmeron-Sanchez, M. The plot thickens: the emerging role of matrix viscosity in cell mechanotransduction. Adv Healthc Mater 9, e1901259, 2020. 31815372 10.1002/adhm.201901259

[54] Caliari, S.R., Vega, S.L., Kwon, M., Soulas, E.M., and Burdick, J.A. Dimensionality and spreading influence MSC YAP/TAZ signaling in hydrogel environments. Biomaterials 103, 314, 2016. 27429252 10.1016/j.biomaterials.2016.06.061 PMC4963302

[55] Major, L.G., Holle, A.W., Young, J.L., *et al.* Volume adaptation controls stem cell mechanotransduction. ACS Appl Mater Interfaces 11, 45520, 2019. 31714734 10.1021/acsami.9b19770

[56] Mattei, G., Cacopardo, L., and Ahluwalia, A. Engineering gels with time—evolving viscoelasticity. Materials 13, 1, 2020. 10.3390/ma13020438 PMC7014018 31963333

[57] Chaudhuri, O., Gu, L., Darnell, M., *et al.* Substrate stress relaxation regulates cell spreading. Nat Commun 6, 1, 2015. 10.1038/ncomms7365 PMC4518451 25695512

[58] Lou, J., Stowers, R., Nam, S., Xia, Y., and Chaudhuri, O. Stress relaxing hyaluronic acid-collagen hydrogels promote cell spreading, fiber remodeling, and focal adhesion formation in 3D cell culture. Biomaterials 154, 213, 2018. 29132046 10.1016/j.biomaterials.2017.11.004

[59] Rodell, C.B., MacArthur, J.W., Dorsey, S.M., *et al.* Shear-thinning supramolecular hydrogels with secondary autonomous covalent crosslinking to modulate viscoelastic properties in vivo. Adv Funct Mater 25, 636, 2015. 26526097 10.1002/adfm.201403550 PMC4624407

[60] Guvendiren, M., and Burdick, J.A. Stiffening hydrogels to probe short- and long-term cellular responses to dynamic mechanics. Nat Commun **3,** 792, 2012. 10.1038/ncomms1792 22531177

[61] Rosales, A.M., Vega, S.L., DelRio, F.W., Burdick, J.A., and Anseth, K.S. Hydrogels with reversible mechanics to probe dynamic cell microenvironments. Angew Chemie Int Ed Engl 56, 12132, 2017. 10.1002/anie.201705684 PMC5668133 28799225

[62] Tran, K.A., Kraus, E., Clark, A.T., *et al.* Dynamic tuning of viscoelastic hydrogels with carbonyl iron microparticles reveals the rapid response of cells to three-dimensional substrate mechanics. ACS Appl Mater Interfaces 13, 20947, 2021. 33909398 10.1021/acsami.0c21868 PMC8317442

[63] Legerstee, K., Geverts, B., Slotman, J.A., and Houtsmuller, A.B. Dynamics and distribution of paxillin, vinculin, zyxin and VASP depend on focal adhesion location and orientation. Sci Rep 9, 1, 2019. 31320676 10.1038/s41598-019-46905-2 PMC6639384

[64] Kuo, C.W., Chueh, D.Y., and Chen, P. Investigation of size-dependent cell adhesion on nanostructured interfaces. J Nanobiotechnology 12, 1, 2014. 25477150 10.1186/s12951-014-0054-4 PMC4265325

[65] Stehbens, S.J., and Wittmann, T. Analysis of focal adhesion turnover Methods Cell Biol **123,** 335, 2014. 10.1016/B978-0-12-420138-5.00018-5 PMC4198331 24974036

[66] Kim, D.H., and Wirtz, D. Predicting how cells spread and migrate: focal adhesion size does matter. Cell Adhes Migr 7, 293, 2013. 10.4161/cam.24804 PMC3711996 23628962

[67] Elosegui-Artola, A. The extracellular matrix viscoelasticity as a regulator of cell and tissue dynamics. Curr Opin Cell Biol 72, 10, 2021. 33993058 10.1016/j.ceb.2021.04.002

[68] Gong, Z., Szczesny, S.E., Caliari, S.R., *et al.* Matching material and cellular timescales maximizes cell spreading on viscoelastic substrates. Proc Natl Acad Sci U S A 115, E2686, 2018. 29507238 10.1073/pnas.1716620115 PMC5866566

[69] De, P.S., and De, R. Stick-slip dynamics of migrating cells on viscoelastic substrates. Phys Rev E 100, 012409, 2019. 31499904 10.1103/PhysRevE.100.012409

[70] Lindstedt, S.L., and Calder, W.A. hoofed in even and odd-toed richness and parasite Host traits species and Perissodactyla mammals, Artiodactyla. Q Rev Biol 56, 1, 1981.

[71] Costello, C., Rossenu, S., Vermeulen, A., Cleton, A., and Dunne, A. A time scaling approach to develop an in vitro-in vivo correlation (IVIVC) model using a convolution-based technique. J Pharmacokinet Pharmacodyn 38, 519, 2011. 21735135 10.1007/s10928-011-9206-4

[72] Brockmeier, D., Dengler, H.J., and Voegele, D. In vitro–in vivo correlation of dissolution, a time scaling problem? Transformation of in vitro results to the in vivo situation, using theophylline as a practical example. Eur J Clin Pharmacol 28, 291, 1985. 4007033 10.1007/BF00543326

[73] Ahluwalia, A. Allometric scaling in-vitro. Sci Rep 7, 42113, 2017. 28169362 10.1038/srep42113 PMC5294453

[74] Magliaro, C., Rinaldo, A., and Ahluwalia, A. Allometric Scaling of physiologically-relevant organoids. Sci Rep 9, 1, 2019. 31417119 10.1038/s41598-019-48347-2 PMC6695443

[75] Mathur, J., Shenoy, V., and Pathak, A. Mechanical memory in cells emerges from mechanotransduction with transcriptional feedback and epigenetic plasticity. bioRxiv, 1, 2020.

[76] Nemec, S., and Kilian, K.A. Materials control of the epigenetics underlying cell plasticity. Nat Rev Mater 6, 69,2021.

[77] Balestrini, J.L., Chaudhry, S., Sarrazy, V., Koehler, A., and Hinz, B. The mechanical memory of lung myofibroblasts. Integr Biol 4, 410, 2012. 10.1039/c2ib00149g 22410748

[78] Yang, C., Tibbitt, M.W., Basta, L., and Anseth, K.S. Mechanical memory and dosing influence stem cell fate. Nat Mater 13, 645, 2014. 24633344 10.1038/nmat3889 PMC4031270

[79] Peng, T., Liu, L., MacLean, A.L., *et al.* A mathematical model of mechanotransduction reveals how mechanical memory regulates mesenchymal stem cell fate decisions. BMC Syst Biol 11, 1, 2017. 28511648 10.1186/s12918-017-0429-x PMC5434622

[80] Alenghat, F.J., and Ingber, D.E. Mechanotransduction: all signals point to cytoskeleton, matrix, and integrins. Sci STKE 2002, pe6, 2002. 11842240 10.1126/stke.2002.119.pe6

[81] Trappmann, B., and Chen, C.S. How cells sense extracellular matrix stiffness: a material's perspective. Curr Opin Biotechnol 24, 948, 2013. 23611564 10.1016/j.copbio.2013.03.020 PMC4037408

[82] Grevesse, T., Versaevel, M., Circelli, G., Desprez, S., and Gabriele, S. A simple route to functionalize polyacrylamide hydrogels for the independent tuning of mechanotransduction cues. Lab Chip 13, 777, 2013. 23334710 10.1039/c2lc41168g

[83] Han, S.J., Bielawski, K.S., Ting, L.H., Rodriguez, M.L., and Sniadecki, N.J. Decoupling substrate stiffness, spread area, and micropost density: a close spatial relationship between traction forces and focal adhesions. Biophys J 103, 640, 2012. 22947925 10.1016/j.bpj.2012.07.023 PMC3443781

[84] Engler, A., Bacakova, L., Newman, C., *et al.* Substrate compliance vs ligand density. Biophys J 86, 1, 2003. 10.1016/S0006-3495(04)74140-5 PMC1303831 14695306

[85] Paul, C.D., Hruska, A., Staunton, J.R., *et al.* Probing cellular response to topography in three dimensions. Biomaterials 197, 101, 2019. 30641262 10.1016/j.biomaterials.2019.01.009 PMC6390976

[86] Mattei, G., Ferretti, C., Tirella, A., Ahluwalia, A., and Mattioli-Belmonte, M. Decoupling the role of stiffness from other hydroxyapatite signalling cues in periosteal derived stem cell differentiation. Sci Rep 5, 1, 2015. 10.1038/srep10778 PMC4451686 26035412

[87] Lin, C.C., and Anseth, K.S. PEG hydrogels for the controlled release of biomolecules in regenerative medicine. Pharm Res 26, 631, 2009. 19089601 10.1007/s11095-008-9801-2 PMC5892412

[88] Vandrangi, P., Gott, S.C., Kozaka, R., Rodgers, V.G.J., and Rao, M.P. Comparative endothelial cell response on topographically patterned titanium and silicon substrates with micrometer to sub-micrometer feature sizes. PLoS One 9, 2014. 10.1371/journal.pone.0111465 PMC4214724 25357245

[89] Carter, D.R., Van Der Meulen, M.C., and Beaupré, G.S. Mechanical factors in bone growth and development. Bone 18, 5S, 1996. 8717541 10.1016/8756-3282(95)00373-8

[90] Wu, M., Fannin, J., Rice, K.M., Wang, B., and Blough, E,R. Effect of aging on cellular mechanotransduction. Ageing Res Rev 10, 1, 2011. 19932197 10.1016/j.arr.2009.11.002 PMC2888727

[91] Sack, I., Beierbach, B., Wuerfel, J., *et al.* The impact of aging and gender on brain viscoelasticity. Neuroimage Elsevier Inc., 46, 652, 2009. 10.1016/j.neuroimage.2009.02.040 19281851

[92] Escoffier, C., Rigal, J De, and Rochefort, A. Age-Related Mechanical An In Vivo Study. J Invest Dermatol 93, 353, 1989. 2768836

[93] Vogel, H.G. Influence of maturation and aging on mechanical and biochemical properties of connective tissue in rats. Mech Ageing Dev 14, 283, 1980. 7206819 10.1016/0047-6374(80)90002-0

[94] Ebihara, T., Venkatesan, N., Tanaka, R., and Ludwig, M.S. Changes in Extracellular Matrix and Tissue Viscoelasticity in Bleomycin-induced Lung Fibrosis Temporal Aspects [Internet]. J Respir Crit Care Med 162, 1569, 2000. 10.1164/ajrccm.162.4.9912011 11029378

[95] Bedossa, P., and Paradis, V. Liver extracellular matrix in health and disease. J Pathol 200, 504, 2003. 12845618 10.1002/path.1397

[96] McKinnon, D.D., Domaille, D.W., Cha, J.N., and Anseth, K.S. Biophysically defined and cytocompatible covalently adaptable networks as viscoelastic 3d cell culture systems. Adv Mater 26, 865, 2014. 24127293 10.1002/adma.201303680 PMC4582033

