# Supplementary material for: Characterizing and Engineering Biomimetic Materials for Viscoelastic Mechanotransduction Studies
Source: Tissue Eng Part B Rev. 2022 Aug 8;28(4):912–25. doi: 10.1089/ten.teb.2021.0151 (PMC9419958; doi:10.1089/ten.teb.2021.0151)
Supplement: Supplemental data [file Supp_Data.docx]

**Supplementary Information**

**Characterising and engineering biomimetic materials for viscoelastic mechanotransduction studies
Ludovica Cacopardo, Nicole Guazzelli, Arti Ahluwalia**

***SI1 Quasi-linear and non-linear models***

Although we focus on linear viscoelastic models for their simplicity and general applicability to most soft tissues and hydrogels, it behooves us to underline that there are several more complex models which have been used to describe the behaviour of viscoelastic materials. A brief summary is given here, but the interested reader can refer to a number of texts and papers on the topic ^1–6^. The quasi-linear viscoelastic (QLV) model is based on the definition of an elastic and a relaxation function, which considers the stress at the time t as a function of the previous elastic state weighted for the differential of the relaxation function^4–6^. Fractional models consider a spectrum of relaxation times parameterized according to a specific fractional derivative order (α). The numerical approximation of the fractional derivative (e.g. with Prony series or with a Kelvin-Voigt fractional derivative – KVFD – model) may be onerous for non-experts as it requires computational skills. Non-linear models are often based on combinations of dashpots and springs with complex ‘constitutive’ equations. For instance, Xu et al. recently proposed a new non-linear model to describe material thermal transitions in which the viscous element is described by a time dependent power law while the springs can be tuned as a function of strain to account for strain hardening ^7^. Non-linear models are usually too difficult to manage for most experimenters because of the complicated fitting procedures involved.

***SI2 Nomenclature for Lumped Parameter Models***

Figure 1S and Table 2S reports the viscoelastic models for solid and fluid materials (classified according to Table 1S) and their nomenclature in the literature ^8–14^. GM is also known as Maxwell-Wiechert and the GV as the generalised Kelvin-Voigt model ^10,11,14^. In the case of first order models (which have one Maxwell or Voigt arm in series or parallel with a spring), GM and GV are also referred to as standard linear solid (SLS) or Zener models. Viscoelastic fluid models, such as Jeffrey or Burger models ^8,12,15,16^, have been also used for describing hydrogels, although GV or GM elements generally fit better with their behaviour ^17^.

*
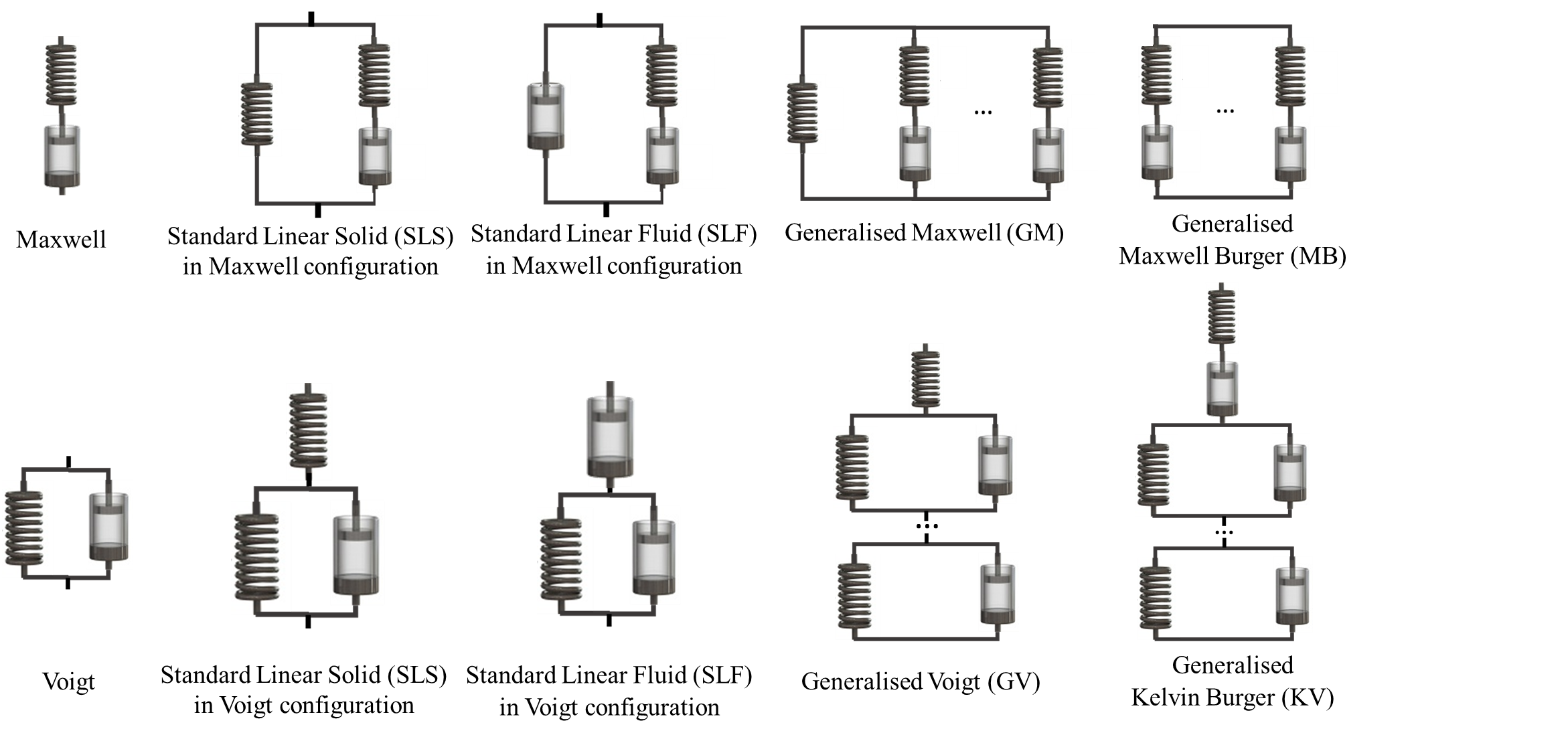
*

*Figure 1S: Viscoelastic models*

Table 1S: Classification of the viscoelastic solid and fluid materials according to their instantaneous and equilibrium responses ^8^

| Material type | Instantaneous compliance ($J_{inst}$) | Equilibrium compliance ($J_{eq}$) | Instantaneous modulus ($E_{inst}$) | Equilibrium modulus ($E_{eq}$) |
| --- | --- | --- | --- | --- |
| Solid | $\geq0$ | $<\infty$ | $\leq\infty$ | $>0$ |
| Fluid | $\geq0$ | $=\infty$ | $\leq\infty$ | $=0$ |

Table 2S: Nomenclature of the main linear viscoelastic models (*see Table 1S, n = model order)

| **Linear model** | **Also known as** | **Number of elements (spring/dashpot)** | **Material type*** |
| --- | --- | --- | --- |
| Voigt | Kelvin-Voigt | 2 | Solid |
| Maxwell | Maxwell-Weichert | 2 | Fluid |
| Standard Linear Solid (SLS)  in Maxwell configuration | Zener model in Maxwell configuration | 3 | Solid |
| Standard Linear Solid (SLS)  in Voigt configuration | Zener model in Voigt configuration | 3 | Solid |
| Standard Linear Fluid (SLF)  in Maxwell configuration | Anti-Zener or Jeffrey’s model  in Maxwell configuration | 3 | Fluid |
| Standard Linear Fluid (SLF)  in Voigt configuration | Anti-Zener or Jeffrey’s model  In Voigt configuration | 3 | Fluid |
| Generalised Voigt (GV) | Generalised Kelvin, Generalised Kelvin-Voigt, Generalised Voigt for solid materials | n+2 | Solid |
| Generalised Maxwell (GM) | Generalised Maxwell-Weichert, Generalised Maxwell for solid materials | n+2 | Solid |
| Generalised Kelvin Burger (KB) | Generalised Voigt for fluid materials, Burger model in Voigt configuration (if n=1) | n+3 | Fluid |
| Generalised Maxwell Burger (MB) | Generalised Maxwell for fluid materials,  Burger model in Maxwell configuration (if n=1) | n+3 | Fluid |

***SI3 Measuring Viscoelasticity***

*Bulk and local mechanical properties*

Material elastic properties are generally derived by analysing the stress-strain curve resulting from tensile or compressive tests. Bulk tests performed with universal testing machines give information on volumetric properties on the scale of a few millimetres or centimetres that can be considered an ‘average’ of the mechanical properties of the material. Micro or nano-structured materials can be probed using AFM or nanoindentation, at respectively the nano and microscale which may be more appropriate to correlate cell response with local mechanical properties^18,19^.. The interested reader may refer to several excellent reviews on the topic ^20–26^. It should be noted that current methods of measurement at these scales are based on the use of indenters or markers and a number of a priori assumptions (e.g., relationship between indenter shape and strain or contact area, elastic behaviour) are necessary for the derivation of viscoelastic parameters which are meaningful and of comparative value. Moreover, measurement reproducibility largely depends on the homogeneity of the material surface: a high surface roughness or an intrinsic material anisotropy will inevitably result in high variability of data. Another consideration to bear in mind is that hydrogels are often prepared by exposing the sample surface to a chemical or photo-crosslinking agent. As the reagent or light penetrates the sample it creates a gradient that may also be reflected in the local mechanical properties ^27,28^. For these reasons bulk mechanical properties are generally more reliable in the case of soft materials. Table 2S summarises the spatial and time resolution of the most common testing methods.

*Table 2S: The spatial and time resolution of mechanical testing methods*

| **Method** | **Spatial Resolution** | **Time Resolution** | **Ref** |
| --- | --- | --- | --- |
| AFM | nanometres | nanoseconds - milliseconds | ^29–31^ |
| Nanoindentation | micrometres | microseconds - seconds | ^32–35^ |
| Bulk tests | millimetres-centimetres | milliseconds - seconds | ^36,37^ |

*Testing methods*

Creep and stress relaxation (SR) tests are the most common viscoelastic testing methods. They are based on the application of a strain or stress step input and on the measurement of the stress or strain response of the material over time respectively ^38^. For an ideal elastic material, the response is a constant stress/strain that is maintained as long as the stimulus is applied. On the contrary, in a viscoelastic material, the initial stress/strain changes over time and in most cases an equilibrium state is reached after some delay ^39^. The material’s parameters and descriptors are generally obtained by fitting exponentials to the time responses. Although simple to perform, the main disadvantages of these tests are that an ideal step is never physically implementable and that they require a priori knowledge of the material’s LVR to truncate data pertaining to non-linear regions ^40,41^. These issues are overcome with the epsilon- and the sigma-dot methods ($\dot{\varepsilon}M$ or $\dot{\sigma}M$), which are based on strain or stress ramps at different constant strain or stress rates. Using this approach, a set of stress- or strain-time curves with different slopes is obtained and the model parameters can be derived through global fitting with shared parameters. The advantages of ramp-based methods are the faithful application of input stimuli and the possibility of testing samples within their LVR. However, a discrete and limited number of strain-rates can be applied and investigating material response to low-strain rates may be time-consuming ^14,41^.

As viscoelastic materials are time-dependent, their response naturally depends also on frequency. In dynamic mechanical analysis (DMA), the change in amplitude and phase between an applied sinusoidal stimulus and corresponding material response is measured as a function of the frequency. Both tensile, compressive and shear properties can be investigated DMA’s advantages include the possibility of investigating material behaviour over a wide range of frequencies, although sweeping through the lower frequencies may be time consuming ^42–44^. In analogy with the frequency spectrum used in DMA, the $\dot{\varepsilon}$- and $\dot{\sigma}$-spectrum are respectively based on the definition of the apparent elastic modulus and apparent compliance spectrum (E_app_($\dot{\varepsilon}$) and J_app_($\dot{\sigma}$)). These functions can be used to derive the model parameters from a single curve fitting, resulting in a lower computational time with respect to other ramp-based tests, but also in a lower precision (i.e. higher estimation error related to dataset compression) ^14,45^.

Table 3S summarises the main testing methods and their basic equations in the time and Laplace domain.

Table 3S: The main viscoelastic testing methods

| INPUT | TIME DOMAIN | LAPLACE DOMAIN | TEST | TYPICAL DURATION/FREQUENCY | REF. |
| --- | --- | --- | --- | --- | --- |
| Step | ε(t) = ε_0_ | ε(s) = ε_0_/s | Stress-relaxation (SR) | 15-60 min | ^38^ |
|  | σ(t) = σ_0_ | σ(s) = σ_0_/s | Creep | 15-60 min | ^38^ |
| Ramp | ε(t) = $\dot{\varepsilon}$t | ε(s) = $\dot{\varepsilon}$/s^2^ | Epsilon-dot ($\dot{\varepsilon})$method/spectrum | 1-30 minutes | ^41,45,46^ |
|  | σ(t) = $\dot{\sigma}$t | σ(s) = $\dot{\sigma}$/s^2^ | Sigma-dot ($\dot{\sigma})$method/spectrum | 1-30 min | ^14^ |
| Sinusoid | ε(t) = ε_0_sin(ωt) | ε(s) = ε_0_ω/s^2^+ω^2^ | Dynamic Mechanical Analysis (DMA) | 10^-2^ – 10^2^ Hz | ^42^ |
|  | σ(t) = σ_0_sin(ωt) | σ(s) = σ_0_ω/s^2^+ω^2^ | DMA | 10^-2^ – 10^2^ Hz | ^42^ |

***SI4 Lumped parameter constitutive equations***

The transfer functions in the Laplace domain of the main linear models for solid and fluid materials, representing the model elasticity - E (s) or compliance - J (s), are reported in Table 4S. Substituting the Laplace transform of the input stimuli in the transfer function and applying the inverse transform, one obtains the model response over time for a given input. As an example, Table 5S reports the time-response of the models for a step input. The time-responses for different strain inputs and the complex modulus expression for the GM model are shown in Table 6S.

Table 4S: Transfer functions in the Laplace domain for solid and liquid materials

| Solid | GM Model | $E\left( s \right)=\frac{\sigma\left( s \right)}{\varepsilon\left( s \right)}= \left( E_{M0}+\sum_{i=1}^{n} \frac{E_{Mi}\eta_{Mi}s}{E_{Mi}+\eta_{Mi}s} \right)$ |
| --- | --- | --- |
|  | GV Model | $J\left( s \right)=\frac{\varepsilon\left( s \right)}{\sigma\left( s \right)}= \left( \frac{1}{E_{V0}}+\sum_{i=1}^{n} \frac{1}{E_{Vi}+\eta_{Vi}s} \right)$ |
| Fluid | MB Model | $E\left( s \right)=\frac{\sigma\left( s \right)}{\varepsilon\left( s \right)}= \left( \sum_{i=0}^{n} \frac{E_{Mi}\eta_{Mi}s}{E_{Mi}+\eta_{Mi}s} \right)$ |
|  | KB Model | $J\left( s \right)=\frac{\varepsilon\left( s \right)}{\sigma\left( s \right)}= \left( \frac{1}{E_{V0}}+\frac{1}{\eta_{V0}s}+\sum_{i=1}^{n} \frac{1}{E_{Vi}+\eta_{Vi}s} \right)$ |

Table 5S: Time responses for a step input

|  |  | input | | Time-response |
| --- | --- | --- | --- | --- |
| Solid | GM Model | ε(t) = ε_0_ | ε(s) = ε_0_/s | $\sigma\left( t \right)= \varepsilon_{0}\left[ E_{M0}+ \sum_{i=1}^{n} E_{Mi}e^{-\frac{E_{Mi}}{\eta_{Mi}}t} \right]$ |
|  | GV Model | σ(t) = σ_0_ | σ(s) = σ_0_/s | $\varepsilon\left( t \right)= \sigma_{0}\left[ \frac{1}{E_{V0}}+ \sum_{i=1}^{n} \frac{1}{E_{Vi}}\left( 1-e^{-\frac{E_{Vi}}{\eta_{Vi}}t} \right) \right]$ |
| Fluid | MB Model | ε(t) = ε_0_ | ε(s) = ε_0_/s | $\sigma\left( t \right)= \varepsilon_{0}\left[ \sum_{i=0}^{n} E_{Mi}e^{-\frac{E_{Mi}}{\eta_{Mi}}t} \right]$ |
|  | KB Model | σ(t) = σ_0_ | σ(s) = σ_0_/s | $\varepsilon\left( t \right)= \sigma_{0}\left[ \frac{1}{E_{V0}}+\frac{t}{\eta_{V0}}+ \sum_{i=1}^{n} \frac{1}{E_{Vi}}\left( 1-e^{-\frac{E_{Vi}}{\eta_{Vi}}t} \right) \right]$ |

| INPUT | | | GM model time/frequency response |
| --- | --- | --- | --- |
| Step strain | ε(t) = ε_0_ | ε(s) = ε_0_/s | $\sigma\left( t \right)= \varepsilon_{0}\left[ E_{M0}+ \sum_{i=1}^{n} E_{\mathrm{Mi}}e^{-\frac{E_{\mathrm{Mi}}}{\eta_{\mathrm{Mi}}}t} \right]$ |
| Step stress | $\sigma$ (t) = $\sigma$ _0_ | $\sigma$ (s) = $\sigma$_0_/s | $\varepsilon\left( t \right)= \sigma_{0}\left[ \frac{1}{E_{M0}}- \sum_{i=1}^{n} \frac{E_{\mathrm{Mi}}}{E_{M0}\left( E_{M0}+E_{\mathrm{Mi}} \right)}\left( e^{-\frac{E_{\mathrm{Mi}}E_{M0}}{\left( E_{M0}+E_{\mathrm{Mi}} \right)\eta_{\mathrm{Mi}}} t} \right) \right]$ |
| Ramp strain (constant strain rate) | ε(t) = $\dot{\varepsilon}$t | ε(s) = $\dot{\varepsilon}$/s^2^ | $\sigma\left( t \right)= \dot{\varepsilon}\left[ E_{M0}t+ \sum_{i=1}^{n} \eta_{\mathrm{Mi}}\left( 1-E_{\mathrm{Mi}}e^{-\frac{E_{\mathrm{Mi}}}{\eta_{\mathrm{Mi}}}t} \right) \right]$ |
| Ramp strain (constant strain rate) | $\sigma$(t) = $\dot{\sigma}$t | $\sigma$(s) = $\dot{\sigma}$/s^2^ | $\varepsilon\left( t \right)= \dot{\sigma}\left[ \frac{t}{E_{M0}}+ \sum_{i=1}^{n} \frac{\eta_{\mathrm{Mi}}}{{E_{M0}}^{2}}\left( 1-e^{-\frac{E_{\mathrm{Mi}}E_{M0}}{\left( E_{M0}+E_{\mathrm{Mi}} \right)\eta_{\mathrm{Mi}}} t} \right) \right]$ |
| Sinusoidal strain | ε(t) = ε_0_sin(ωt) | ε(s) = ε_0_ω/s^2^+ω^2^ | * |

*Table 6S: Generalised Maxwell (GM) model (for solids) response for different inputs*

* The complex modulus is more common. Here s=jω, giving:

$$E^{*}\left( \omega\right)= \left( E_{M0}+\sum_{i=1}^{n} \frac{E_{\mathrm{Mi}}\omega^{2}}{{{(E}_{\mathrm{Mi}}/\eta_{Mi})}^{2}+\omega^{2}} \right)+ j\left( \sum_{i=1}^{n} \frac{{{(E}_{\mathrm{Mi}}}^{2}{/\eta}_{Mi})\omega}{{{(E}_{\mathrm{Mi}}/\eta_{Mi})}^{2}+\omega^{2}} \right)$$

where the first terms is the storage modulus E’ and the second term, associated with the imaginary part of the modulus, is the loss modulus E’’.

E’ and E’’ can be also derived from the strain-rate domain descriptor. As an example, considering the instantaneous and equilibrium moduli (E_inst_, E_eq_) and the characteristic relaxation time τ_relax_ derived from a first-order General Maxwell model, E’ and E’’ can be calculated as follow:

$E’= E_{\mathrm{eq}}+\frac{\left( E_{\mathrm{inst}}- E_{\mathrm{eq}} \right)\omega^{2}\tau^{2}}{1+ \omega^{2}\tau^{2}}$;

$E’’= \frac{\left( E_{\mathrm{inst}}- E_{\mathrm{eq}} \right) \omega\tau}{1+ \omega^{2}\tau^{2}}$.

***SI5 Calculation and Equivalence of viscoelastic descriptors for first order GM and GV models***

The viscoelastic descriptors, i.e. the instantaneous (E_inst_) and equilibrium elastic moduli (E_eq_) and the characteristic times (τ_i_), can be calculated from the lumped model parameters (Figure 2S). An overview of the viscoelastic descriptors for the GM and GV model is shown Table 7S.


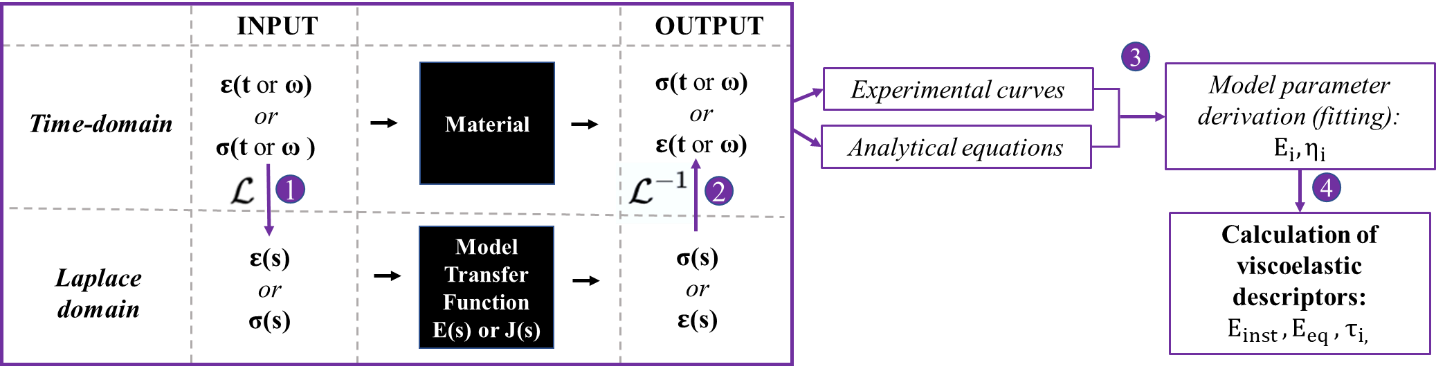


*Figure 2S: Workflow for identifying material viscoelastic properties:1) Laplace transform of the experimental input stimuli; 2) Laplace anti-transform of the model output; 3) Model parameters are derived by fitting the analytical expression of the model output in the time or frequency domain to the experimental stress- or strain-time or frequency data; 4) Viscoelastic descriptors are calculated using the derived parameters. Further theoretical details can be found in SI3-4.*

Table 7S: Viscoelastic descriptors as a function of the GM and GV model parameters


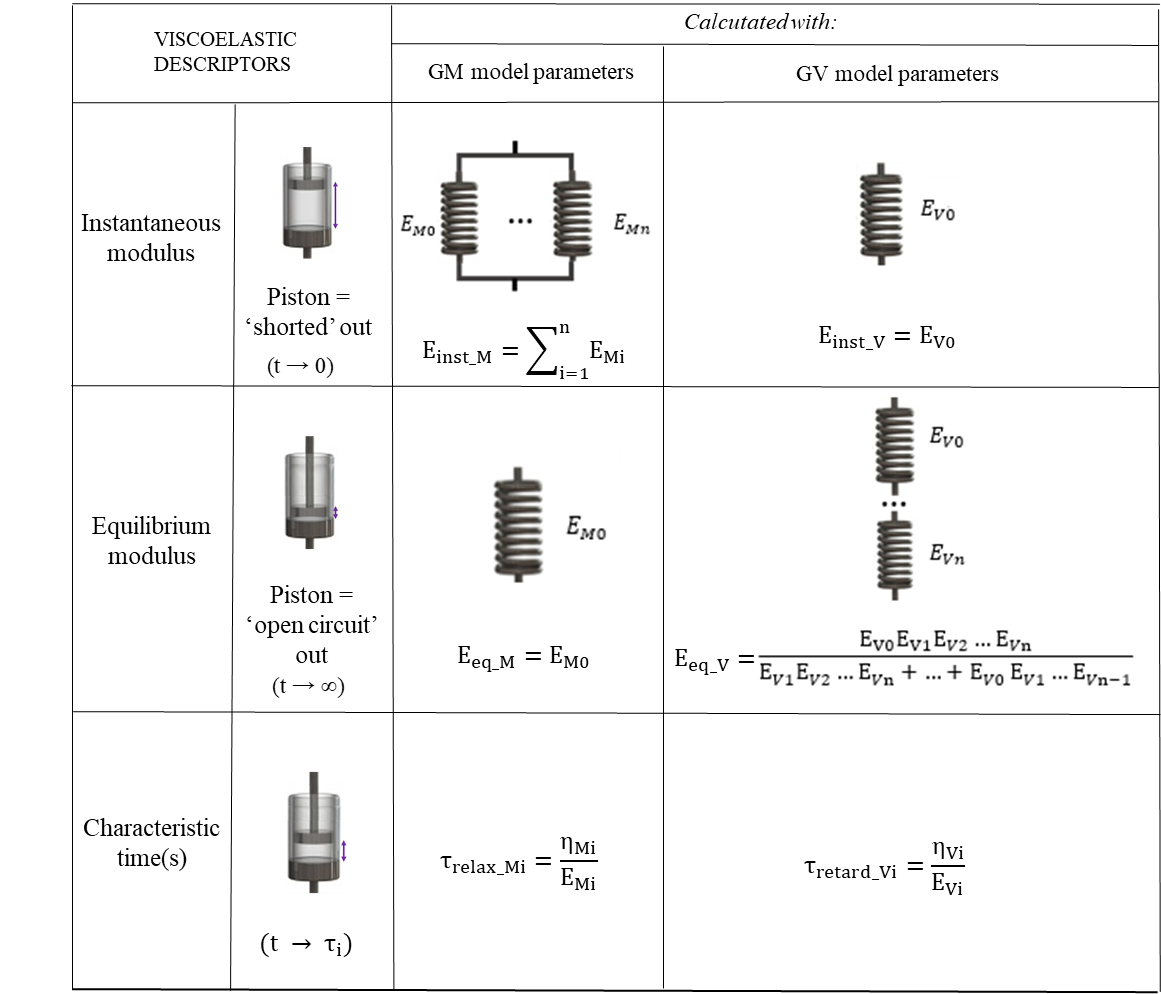


The GV and GM model parameters can be reciprocally derived from the equivalence of the viscoelastic descriptors.

In complex or composite materials, such as gels, the different τ_i_ are often attributed to the phenomena (e.g. polymeric chain sliding, liquid phase flow, etc.) which contribute to the overall material viscous behaviour with different characteristic times ^47–49^. As most of these phenomena have overlapping times and are often cooperative or related in some way, a single characteristic time, as obtained with first order (n=1) models, is usually enough to describe material viscoelastic behaviour. It also avoids over-parameterization and over-interpretation ^47–50^.

Considering first order models and imposing the equivalence of E_inst_, E_eq_ and τ obtained with the two models:

$E_{V0}= E_{M0}+E_{M1}$ ;

$E_{V1}= E_{M0}\left( 1+ \frac{E_{M0}}{E_{M1}} \right)$;

$\eta_{V1}$= ${\eta_{M1}\left( 1+ \frac{E_{M0}}{E_{M1}} \right)}^{2}$;

$E_{M0}=\frac{E_{V0}E_{V1}}{E_{V0}+E_{V1}}$ ;

$E_{M1}=\frac{\left( E_{V0} \right)^{2}}{E_{V0}+E_{V1}}$ ;

$\eta_{M1}$= $\eta_{V1}\left( \frac{E_{V0}}{E_{V0}+E_{V1}} \right)^{2}$.

**SI6 Relationship between characteristic relaxation and retardation times**

Mathematical relationships between the two characteristic times for a GM model (respectively $\tau_{relax\_Mi}\mathrm{and}\tau_{retard\_Mi)}$ can be derived as follows:

- in the case of a strain input (ε_0_),$\tau_{relax\_Mi}$ $=\frac{\eta_{Mi}}{E_{Mi}}$;
- in the case of a stress input (σ_0_), $\tau_{retard\_Mi}$ $=\eta_{Mi}\left( \frac{E_{M0}+{{E_{\mathrm{Mi}}}}}{E_{M0}E_{Mi}} \right).$

Their ratio can thus be expressed as:

$\frac{\tau_{relax\_Mi}}{\tau_{retard\_Mi}} =\frac{E_{M0}}{{E_{M0}+E_{\mathrm{Mi}}}}$.

For first order models, the ratio of the GM equilibrium and instantaneous elastic modulus (respectively $E_{eq\_M}\mathrm{and}E_{\mathrm{inst}{\_}_{M}}$):

$\frac{\tau_{relax\_Mi}}{\tau_{retard\_Mi}}=\frac{E_{M0}}{{E_{M0}+E_{M1}}}=\frac{E_{eq\_M}}{E_{inst\_M}}$.

Similarly, for the GV model:

- in the case of a strain input (ε_0_),$\tau_{relax\_Vi}$ $=\frac{\eta_{Vi}}{{(E_{V0}+E_{Vi})}}$;
- in the case of a stress input (σ_0_), $\tau_{retard\_Vi}$ $=\frac{\eta_{Vi}}{E_{Vi}} .$

$\frac{\tau_{relax\_Vi}}{\tau_{retard\_Vi}}=\frac{E_{\mathrm{Vi}}}{\left( E_{V0}+E_{\mathrm{Vi}} \right)}$.

For first order models, the ratio of the GV equilibrium and instantaneous elastic modulus (respectively $E_{eq\_V}\mathrm{and}E_{\mathrm{inst}{\_}_{V}}$):

$\frac{\tau_{relax\_Vi}}{\tau_{retard\_Vi}}=\frac{E_{V1}}{\left( E_{V0}+E_{V1} \right)}=\frac{E_{eq\_V}}{E_{inst\_V}}$.

These relationships are useful when comparing data from studies which use different mechanical inputs (i.e., stress as opposed to strain inputs or vice versa).

***References***

1. Schapery RA: An engineering theory of nonlinear viscoelasticity with applications. Int J Solids Struct Pergamon Press Ltd., 1966; 2:407–25: http://dx.doi.org/10.1016/0020-7683(66)90030-8.

2. Nekouzadeh A, Pryse KM, Elson EL, and Genin GM: A simplified approach to quasi-linear viscoelastic modeling. J Biomech 2007; 40:3070–8.

3. Haslach HW: Nonlinear viscoelastic, thermodynamically consistent, models for biological soft tissue. Biomech Model Mechanobiol 2005; 3:172–89.

4. Zhang W, Capilnasiu A, Sommer G, Holzapfel GA, and Nordsletten DA: An efficient and accurate method for modeling nonlinear fractional viscoelastic biomaterials. Comput Methods Appl Mech Eng Elsevier B.V., 2020; 362:112834: https://doi.org/10.1016/j.cma.2020.112834.

5. Feng Z, Kosawada T, Nakamura T, et al.: Theoretical methods and models for mechanical properties of soft biomaterials. AIMS Mater Sci 2017; 4:680–705.

6. Mattei G and Ahluwalia A: Sample, testing and analysis variables affecting liver mechanical properties: A review. Acta Biomater 2016; 45:60–71: http://linkinghub.elsevier.com/retrieve/pii/S1742706116304603.

7. Xu Q, Engquist B, Solaimanian M, and Yan K: A new nonlinear viscoelastic model and mathematical solution of solids for improving prediction accuracy. Sci Rep 2020; 10:1–10.

8. Mainardi F and Spada G: Creep, relaxation and viscosity properties for basic fractional models in rheology. Eur Phys J Spec Top 2011; 193:133–60.

9. Roylance D: Engineering viscoelasticity. Dep. Mater. Sci. Eng. Inst. Technol. Cambridge MA. 2001.;

10. Dooling PJ, Buckley CP, and Hinduja S: An intermediate model method for obtaining a discrete relaxation spectrum from creep data. Rheol Acta 1997; 36:472–82.

11. Zaremba LS and Smoleński WH: Optimal portfolio choice under a liability constraint. Ann Oper Res 2000; 97:131–41.

12. Kahshan M, Lu D, and Siddiqui AM: A Jeffrey Fluid Model for a Porous-walled Channel: Application to Flat Plate Dialyzer. Sci Rep 2019; 9:1–18.

13. Serra-Aguila A, Puigoriol-Forcada JM, Reyes G, and Menacho J: Viscoelastic models revisited: characteristics and interconversion formulas for generalized Kelvin–Voigt and Maxwell models. Acta Mech Sin Xuebao The Chinese Society of Theoretical and Applied Mechanics; Institute of Mechanics, Chinese Academy of Sciences, 2019; 35:1191–209: https://doi.org/10.1007/s10409-019-00895-6.

14. Cacopardo L, Mattei G, and Ahluwalia A: A new load-controlled testing method for viscoelastic characterisation through stress-rate measurements. Materialia 2020; 9:100552: https://linkinghub.elsevier.com/retrieve/pii/S2589152919303485.

15. Dogan M, Kayacier A, Toker ÖS, Yilmaz MT, and Karaman S: Steady, Dynamic, Creep, and Recovery Analysis of Ice Cream Mixes Added with Different Concentrations of Xanthan Gum. Food Bioprocess Technol 2013; 6:1420–33.

16. Cooper RF: Seismic wave attenuation: Energy dissipation in viscoelastic crystalline solids. In: Wenk, K. and H.-R., ed. Rev Mineral Geochemistry Mineralogical Scoiety of America, 2002.;

17. Chaudhuri O, Gu L, Darnell M, et al.: Substrate stress relaxation regulates cell spreading. Nat Commun Nature Publishing Group, 2015; 6:1–7: http://dx.doi.org/10.1038/ncomms7365.

18. Basoli F, Giannitelli SM, Gori M, et al.: Biomechanical characterization at the cell scale: Present and prospects. Front Physiol 2018; 9:1–21.

19. Kuo CW, Chueh DY, and Chen P: Investigation of size-dependent cell adhesion on nanostructured interfaces. J Nanobiotechnology 2014; 12:1–10.

20. Ebenstein DM and Pruitt LA: Nanoindentation of soft hydrated materials for application to vascular tissues. 2004;

21. Buffinton CM, Tong KJ, Blaho RA, Buffinton EM, and Ebenstein DM: Comparison of mechanical testing methods for biomaterials: Pipette aspiration, nanoindentation, and macroscale testing. J Mech Behav Biomed Mater Elsevier, 2015; 51:367–79: http://dx.doi.org/10.1016/j.jmbbm.2015.07.022.

22. Rettler E, Hoeppener S, Sigusch BW, and Schubert US: Mapping the mechanical properties of biomaterials on different length scales: Depth-sensing indentation and AFM based nanoindentation. J Mater Chem B 2013; 1:2789–806.

23. Attard P: Measurement and interpretation of elastic and viscoelastic properties with the atomic force microscope. J Phys Condens Matter 2007; 19.

24. Cohen SR and Kalfon-Cohen E: Dynamic nanoindentation by instrumented nanoindentation and force microscopy: A comparative review. Beilstein J Nanotechnol 2013; 4:815–33.

25. Chen DL, Yang PF, and Lai YS: A review of three-dimensional viscoelastic models with an application to viscoelasticity characterization using nanoindentation. Microelectron Reliab Elsevier Ltd, 2012; 52:541–58: http://dx.doi.org/10.1016/j.microrel.2011.10.001.

26. Gibson RF: A review of recent research on nanoindentation of polymer composites and their constituents. Compos Sci Technol 2014; 105:51–65.

27. Hajikhani A, Scocozza F, Conti M, et al.: Stochastic modeling for hysteretic bit–rock interaction of a drill string under torsional vibrations. Int J Artif organ 2019; 10:548–57.

28. Cacopardo L, Mattei G, and Ahluwalia A: Alginate gels with spatially tunable mechanical properties for pathophysiological in-vitro models. Proc 23rd Congr Eur Soc Biomech 2017; 15978.

29. Anwar M and Rousso I: Atomic force microscopy with time resolution of microseconds. Appl Phys Lett 2005; 86:1–3.

30. Giridharagopal R, Rayermann GE, Shao G, et al.: Submicrosecond time resolution atomic force microscopy for probing nanoscale dynamics. Nano Lett 2012; 12:893–8.

31. Plodinec M, Loparic M, and Aebi U: Atomic force microscopy for biological imaging and mechanical testing across length scales. Cold Spring Harb Protoc 2010; 5.

32. Optics11: Optics11_PiumaV3_specifications_2020 [Internet]. : Optics11_PiumaV3_specifications_2020.pdf.

33. KLA-tencor: iNano Nanoindentation | Indentation Hardness | KLA [Internet]. : iNano Nanoindentation %7C Indentation Hardness %7C KLA.

34. Minnesota U of: NanoIndenter XP microprobe system | Biomedical Engineering. : NanoIndenter XP microprobe system %7C Biomedical Engineering.

35. Herbert EG, Oliver WC, and Pharr GM: Nanoindentation and the dynamic characterization of viscoelastic solids. J Phys D Appl Phys 2008; 41.

36. Shepherd TN, Zhang J, Ovaert TC, Roeder RK, and Niebur GL: Direct comparison of nanoindentation and macroscopic measurements of bone viscoelasticity. J Mech Behav Biomed Mater Elsevier Ltd, 2011; 4:2055–62: http://dx.doi.org/10.1016/j.jmbbm.2011.07.004.

37. Zwich/Roell: Product Information Materials Testing Machines AllroundLine Z005 to Z100. .

38. Mow VC, Kuei SC, Lai WM, and Armstrong CG: Biphasic Creep and Stress Relaxation of Articular Cartilage in Compression: Theory and Experiments. J Biomech Eng 1980; 102:73: http://biomechanical.asmedigitalcollection.asme.org/article.aspx?articleid=1394848.

39. Selfe J: Fundamentals of Biomechanics. Physiology 2000; 86:163: http://linkinghub.elsevier.com/retrieve/pii/S0031940605611765.

40. Mattei G, Tirella A, Gallone G, and Ahluwalia A: Viscoelastic characterisation of pig liver in unconfined compression. J Biomech 2014; 47:2641–6.

41. Tirella A, Mattei G, and Ahluwalia A: Strain rate viscoelastic analysis of soft and highly hydrated biomaterials. J Biomed Mater Res - Part A 2014; 102A:3352–60.

42. Menard KP: Dynamic Mechanical Analysis. A Practical Introduction. Second edi. Taylor & Francis Group, 2008.;

43. Vodovotz Y, Hallberg L, and Chinachoti ’ P: Effect of Aging and Drying on Thermomechanical Properties of White Bread as Characterized by Dynamic Mechanical Analysis (DMA) and Differential Scanning Calorimetry (DSC). Cereal Chem 1996; 73:264–70: http://www.aaccnet.org/publications/cc/backissues/1996/Documents/73_264.pdf.

44. Mattei G, Tirella A, Gallone G, and Ahluwalia A: Viscoelastic characterisation of pig liver in unconfined compression. J Biomech Elsevier, 2014; 47:2641–6: http://www.jbiomech.com/article/S0021929014003285/fulltext.

45. Mattei G and Ahluwalia A: A new analytical method for estimating lumped parameter constants of linear viscoelastic models from strain rate tests. Mech Time-Dependent Mater The Author(s), 2018; 1–9: http://dx.doi.org/10.1007/s11043-018-9385-0.

46. Mattei G, Gruca G, Rijnveld N, and Ahluwalia a.: The nano-epsilon dot method for strain rate viscoelastic characterisation of soft biomaterials by spherical nano-indentation. J Mech Behav Biomed Mater Elsevier, 2015; 50:150–9: http://linkinghub.elsevier.com/retrieve/pii/S1751616115002088.

47. Darvell BW: Chapter 4 - Rheology. Mater Sci Dent Elsevier, 2018; 92–120.

48. Baurngaertel M, De Rosa ME, Machado J, Masse M, and Winter HH: The relaxation time spectrum of nearly monodisperse polybutadiene melts. Rheol Acta 1992; 31:75–82.

49. Mao R, Tang J, and Swanson BG: Relaxation time spectrum of hydrogels by CONTIN analysis. J Food Sci 2000; 65:374–81.

50. Zhang F, Wang L, Li C, and Xing Y: The Discrete and Continuous Retardation and Relaxation Spectrum Method for Viscoelastic Characterization of Warm Mix Crumb Rubber-Modified Asphalt Mixtures. Materials (Basel) 2020; 13:3723.
